# The contribution of assets to adaptation to extreme temperatures among older adults

**DOI:** 10.1371/journal.pone.0208121

**Published:** 2018-11-29

**Authors:** Ana Raquel Nunes

**Affiliations:** Warwick Medical School, University of Warwick, Coventry, United Kingdom; University of Malaya, MALAYSIA

## Abstract

**Background:**

Climate change and extreme temperatures pose increasing challenges to individuals and their health with older adults being one of the most vulnerable groups. The aim of this paper is to better understand the roles that tangible assets (e.g., physical or financial) and intangible assets (e.g., human or social) play in the way older adults adapt to extreme temperatures, the types of adaptive responses they implement, limits and constraints, as well opportunities for better adaptation. Rather than focusing exclusively on extremes of heat, or considering each type of asset in isolation, the important and novel contribution of this paper is to take an integrated and multi-seasonal qualitative and quantitative approach, that conjointly investigates all categories of assets in relation to the adaptations that independently-living older adults make to both extreme heat and extreme cold.

**Methods and findings:**

The paper examines the contribution of assets to adaptation to extreme temperatures among older adults living independently in their homes. An innovative mixed methods study with an inter-seasonal approach was implemented in Lisbon, Portugal with interviews and surveys during summer for extreme heat and winter for extreme cold. The ability of participants to adapt to extreme temperatures was found to be dependent on asset context and diversity, and the dynamics through which extreme temperatures enhanced or reduced the stock of assets available. As a result, participants engaged in activities of assets replacement, exchange or substitutions. Despite this, many participants recognised many constraints and limits to their ability to adapt and protect their health and well-being ranging from reduced income, high energy costs and lack of social networks. Opportunities for improving older adults’ adaptation were found to exist and strategies, action and investment have been identified by older adults which included life-long education, incentives to improve insulation and local activities.

**Conclusions:**

The paper suggests that the implementation of the proposed asset-based approach linking assets and adaptation to extreme temperatures, illustrates the key pathway that individuals, their families and carers, governments, policymakers, researchers and practitioners can follow to ensure effective adaptation and promote health and well-being. Supporting older adults’ adaptation to extreme temperatures is possible and can be complemented with efforts to reduce older adults’ vulnerability and building resilience to extreme temperatures. These findings pose concrete implications for policy and practice, including for example the need for implementation of measures and actions to reduce poverty, reduce energy costs, improve the quality of the housing stock and improve older adults’ social networks.

## 1. Introduction

Extreme temperatures negatively affect human health and well-being with increasing impacts on mortality [1, 2] and morbidity [3, 4] due to significant exposure and vulnerability [5]. Older adults (aged 65 and over), children, chronically ill individuals, pregnant women and individuals living with low income are especially vulnerable to extreme heat and cold [1, 2, 4, 6, 7]. Furthermore, extreme temperatures impact on the ability of individuals to adapt [5] with consequences for their health and well-being [8]. As ‘the process of adjustment to actual or expected climate and its effects’ [9], adaptation ‘seeks to moderate harm or exploit beneficial opportunities’ [9]. As a result, adaptation strategies are used by individuals to reduce the direct and indirect effects of events such as extreme temperatures [3].

Consequently, many European countries (e.g. England, France, Portugal) annually publish Heatwave and Cold Weather Plans, developed by national governments and aimed at health and social care organisations (e.g. hospitals, care homes), local government authorities (e.g. city councils) and other public services (e.g. schools), as well as individuals and communities. Ultimately, these plans aim at protecting individuals from the risks and impacts of extreme temperatures through the distribution of information and advice regarding how to keep well during extreme temperatures via heat-/cold-health alerts.

Research on older adults’ adaptation to extreme temperatures has explored how they adapt and feel about the challenges posed by such events. In most instances, older adults considered their adaptation strategies as typical daily activities not involving preparation [10]. Research has also found that older adults do not feel old or frail [11–13] and do not feel at risk from extreme heat [10, 14–17] or extreme cold [13]. Evidence from research on extreme heat undertaken in Europe, the US and Australia has highlighted that the use of cooling devices (i.e. fans, air conditioning), modifications in the type of clothing (i.e. wearing lighter clothes), food and drink (i.e. eating lighter foods, drinking more fluids), as well as adjustments to daily routines and rhythms are some of the most used adaptation strategies [14–20]. Past experiences of extreme heat as well as having someone to rely on (e.g. social contacts and networks) positively influence older adults’ ability to implement adaptive strategies and behaviours [17]. In contrast, knowledge and costs of operating cooling devices, poor insulation and social isolation hindered their ability to adapt to extreme heat [17].

On the other hand little is known about adaptation to extreme cold, despite findings that older adults found adapting to cold temperatures more challenging than adapting to heat [10, 18]. Similarly to what was found for heat adaptation, older adults’ income, social networks and housing characteristics were found to influence adaptation to extreme cold temperatures [13]. Energy consumption of heating devices is thus a factor that impedes the ability to adapt due to income constraints [12, 21]. Furthermore, social, cultural and financial circumstances have been identified as influencing the way older adults adapt to extreme cold [13, 22–24].

Despite some advances, more knowledge is needed on what determines how older adults adapt and the factors influencing adaptation to extreme temperatures. As a result, more research is needed to understand the way through which they are able to reduce the impacts of extreme temperatures [18].

A better understanding of the factors (i.e. assets or resources) shaping older adults’ adaptation strategies can help unveiling the challenges they face during extreme temperatures. Authors from diverse disciplinary fields (i.e. public health, environmental sciences and sociology) have advocated the benefits of using asset-based approaches and the concept of assets. There are a considerable number of ways in which assets have been defined and operationalised. According to Bebbington [25] in the sociology literature, assets or capitals ‘are not simply resources that people use […] they are assets that give them the capability to be and to act.’ (page 2022 in [25]). Others like the Ford Foundation [26] in an environmental science context define assets as ‘a broad array of resources that enable people and communities to exert control over their lives and to participate in their societies in meaningful and effective ways’ (page 4 in [26]). In the public health context, on the other hand, assets have been defined as ‘any factor (or resource) which enhances the ability of individuals, groups, […] to maintain and sustain health and wellbeing and to help to reduce health inequities.’ (page 18 in [27]).

These definitions consider assets as resources individuals can access to respond to threats and stresses (e.g. extreme temperatures) determining individuals’ ability to adapt [28]. Such definitions have given rise to an array of ways of operationalising the concept of assets but all fall into the so called ‘five-capitals’ model and have many commonalities [25, 29–33]: a) human, financial, physical, social and natural [31, 33]; b) human, produced, cultural, social and natural [25]; c) human, financial, manufactured or physical, social and natural [15, 34, 35]; d) human, financial, physical, social and environmental [36]. A common categorisation of assets is thus one that includes: human; financial; physical; natural/environmental/public or place-based, and; social assets [30–33, 36]. Table 1 summarises the five categories of assets and the different types of assets that can be associated with them according to the literature. This portrays the many commonalities between the categorisations of assets in the sociology, environmental sciences and public health literatures. Additionally, assets include both tangible assets (natural/environmental/public or place-based; physical and financial) and intangible assets (human; social). This thus, makes the concept of assets a well-fitted framework for researching older adults’ adaptation to extreme temperatures. As a result, in this paper, assets are defined as factors or characteristics directly or indirectly available to individuals in anticipating or responding to threats (i.e. extreme temperatures), and categorized as human, financial, physical, social, place-based. This definition and categorisation were developed for this research taking into account the various definitions and the context of this paper. Having such a categorisation of assets will help answer the research goals.

**Table 1:** Summary of categories of assets, types of assets and sources.

Categories of assets	Types of assets	Sources
Human	Education level, skills, knowledge, good health, ability to work, living arrangements, occupation, nutrition status	[25–27, 29–36]
Financial	Income, savings, access to credit, pensions, informal economy, expenses	
Physical	Buildings, type of housing, housing tenure, roads, tools, appliances, machines, terraces, irrigation canals, power lines, affordable energy, water supply, sanitation, telecommunication facilities, transport	
Natural, Environmental, Public or Place-based	Land, atmosphere, water, trees, wild vegetables, wild animals, fisheries stocks, biodiversity, metals, oil and other environmental resources, access to public amenities and services	
Social	Networks, connectedness, membership of groups and associations, marital status, relationships of trust, support, reciprocity and exchanges	

Asset portfolio is a term that has been applied to define access, availability and accumulation of a diverse and complex range of assets (see Table 1) that individuals manage in their lives [25, 28]. Assets can thus help understand risk and inequalities, as well as impacts on health and well-being [33, 37]. The important and novel contribution of this paper is to take an integrated approach by investigating all categories of assets, rather than in isolation as it has been done before.

Unlike previous studies that have narrowly focused on disciplinary perspectives [6, 28], a single extreme temperature event (heat or cold) [18] and a restricted range of specific factors [6], the approach taken here makes a significant, integrated contribution to understanding and knowledge in adaptation to extreme temperatures. It deliberately sought to better our understanding of what shapes adaptation through an innovative research method and empirical findings aiming at influencing policy and practice.

The aim of this paper is to investigate the categories and types of assets older adults’ access and use when adapting to extreme temperatures, constraints and limits to adaptation and opportunities for better adaptation. Finally, the paper discusses its findings within the existing literature and presents options for improving adaptation to extreme temperatures.

## 2. Methods

### 2.1. Case study: Portugal and the city of Lisbon

The city of Lisbon in Portugal was the location of this research, as it accounts for a series of major extreme temperature events with great human health impacts in recent years, especially for older adults [20, 21]. Lisbon is Portugal’s capital and largest city, with a population of 530847 inhabitants of which 26.9% are older adults (65 years and older) [38], has a warm temperate climate with dry and hot summers, and mild winters [39], and regular heatwaves, and sporadic cold snaps. The impacts of extreme heat have resulted in excess mortality and morbidity [40–43] and rises in estimated mortality by 2020 [43] and 2050 [44] are expected. On the other hand, little is known about the impacts of extreme cold temperatures [45, 46].

### 2.2. Sampling

Data were collected within the city of Lisbon wards. Access to participants was gained through local authorities and public or charitable organisations working with older adults living independently in their homes. Participants were selected using the following inclusion criteria: being 65 years or older; living independently in their homes, and; living in one of the city’s wards. A diverse sample of participants was selected to reflect the Census 2011 data (i.e. age, sex, marital status, living arrangements, education level, financial status) and non-probability sampling techniques (i.e. purposeful, convenience, quota) were used to select participants [47, 48]. The recruitment strategy used was deemed likely to discourage the participation of individuals with dementia or cognitive impairment, as it was based on a free and voluntary willingness to participate. There are no reasons to suspect that possible biases affected the sampling procedure. The author contacted older adults in the same period of the year (Summer).

Potential participants were approached and enquired if they were willing to participate in the two phases of the research (Phase 1 during the Summer months and Phase 2 during the Winter months). Participants with sufficient competence and autonomy were given additional information about the research aims and objectives. They were then assessed on their understanding of the research and potential risks and benefits of participating, as well as option to refuse and withdraw from participating at any time. Informed written consent was obtained for all participants. The author made sure participants understood the importance of their participation and truthfulness of their answers for better understanding of what is needed to improve older adults’ ability to adapt to extreme temperatures and inform policy and practice. The fact that the interviews took place in two phases, one in Summer and the other in Winter allowed the author to develop a connection with all participants for better rapport minimising the risk of biases (e.g. social desirability).

The sample size was obtained to ensure theoretical saturation (i.e. until all concepts were well-developed) [48].

### 2.3. Data collection

Primary data was collected using an inter-seasonal approach which involved interviewing participants during the times of the year more likely to experience extreme temperatures (i.e. summer and winter). This allowed participants to better relate their behaviours and responses to such events. Semi-structured interviews were used to explore participants’ assets and their role in adapting to extreme temperatures. Interview protocols included the collection of quantitative information on socio-demographic, tangible assets (financial assets, physical assets, place-based assets) and intangible assets (human assets, social assets) data (Table 2). The categories and types of assets used have been adapted from those in Table 1 to be adequate to the reality of the case study location. Qualitative information on participants’ adaptive strategies during extreme temperatures, characteristics of assets, constraints and limits to adaptation, and opportunities to improve adaptation were also collected.

**Table 2:** Categories and types of assets data collection.

Categories of assets	Types of assets
Human assets	- Living arrangements - Level of education - Occupation - Health status
Financial assets	- Financial situation - Income - Sources of income - Expenses - Savings - Financial difficulties
Physical assets	- Housing type - Floor number - Existence of a lift (elevator) - Age of building - Housing tenure - Satisfaction with house - Living conditions - Appliances/ equipment / goods
Place-based assets	- Access to facilities, amenities and services - Quality of public facilities, amenities and services - Access to green spaces - Heatwave Plan and Cold Weather Plan
Social assets	- Social contacts and networks - Marital status - Social support - Emotional support - Financial support - Social participation - Social activities

Face-to-face interviews were scheduled to take place in a private and neutral familiar location to participants (e.g. local authority office) [49]. Details on the interview protocols in both the original language (Portuguese) and English are available in the supplementary materials (S1 and S2 Files). Interviews were conducted by the author, who has been trained in quantitative, qualitative and mixed methods research. The interviews were spoken in Portuguese, all questions were asked verbally to participants, audio-recorded, transcribed and analysed in Portuguese. Relevant quotes were translated to English by the author.

A total of 52 heat-related interviews (Phase 1: Summer) and 46 follow-up cold-related interviews with the same participants (Phase 2: Winter) were conducted. Phase 1 interviews were implemented first during the summer months and included the collection of quantitative data and heat-related qualitative data. Phase 2 interviews were implemented after Phase 1 interviews during the subsequent winter months and included the collection of cold-related qualitative data.

Phase 1 interviews ranged from 22 minutes to 1 hour and 47 minutes (mean: 55 minutes; total: 48 hours of interviews). Participation rate in Phase 1 of this study was 100% and in Phase 2 was of 88.5%. Of the 52 participants in Phase 1 only six of them (4 females and 2 males) withdrew from participating in Phase 2. Reasons for withdrawal included health problems, inability to travel, being uncontactable and unwilling to participate. Phase 2 interviews ranged from 17 minutes to 2 hours (mean: 37 minutes; total: 29 hours of interviews). The mean age of participants in Phase 1 of the study was 75.1 years (minimum age: 65 years; maximum age: 95 years) and on Phase 2 was 74.8 years (minimum age: 65 years; maximum age: 95 years). Of the 52 participants in the study, 67% were female, 58% lived alone, and 48% were widowed, 29% were married, 8% were divorced and 15% were single.

Ethical approval for the study was obtained from the University of East Anglia, Faculty of Medicine and Health Sciences Research Ethics Committee (Reference 2011/2012–30) and from Universidade de Lisboa, Instituto de Ciências Sociais Ethical Committee.

### 2.4. Data analysis

Integration of quantitative and qualitative data allowed qualitative data to shape how results are presented [50]. Both sets of data are used to validate, interpret and corroborate each other. In order to increase validity of the study, triangulation was used to appraise, cross check and detect patterns and validate findings within and between quantitative and qualitative data [48].

Quantitative data were transferred to a bespoke data entry and analysis tool developed specially for this study in Microsoft Excel. Qualitative data (i.e. transcripts of audio records in Microsoft Word) were imported into NVivo 9 qualitative Coding Software (QSR International). Quantitative data were analysed to obtain sample characteristics through descriptive statistics (frequency counts, means, ranges, and standard deviations). Due to the sample size the quantitative data does not claim to have representativity but rather, it presents good internal validity and transferability due to triangulation between quantitative and qualitative data, and positioning within the literature (see 4. Discussion). Qualitative data were analysed using a hermeneutical phenomenology approach [51]. All transcripts were grouped, coded and analysed using thematic analysis at both the individual and whole sample levels in NVivo 9 qualitative data analysis software (QSR). Initial themes and codes arose from interview transcripts and were refined and changed using an iterative process until final themes and codes were obtained. This involved the coding and categorisation of all data using a systematic approach which enabled data interpretation and the identification of themes and sub-themes.

## 3. Results

The analysis of both quantitative and qualitative data revealed a deep understanding of the drivers influencing older adults’ adaptation to extreme temperatures and the contribution of assets. Descriptive quantitative results for specific instances of the five asset types are presented in the supplementary material (S1 Table). Themes from the qualitative data were coded using the five main categories of assets identified above (Table 2): 1) human assets; 2) financial assets; 3) physical assets; 4) place-based assets; and 5) social assets. The context and diversity of themes will be discussed in detail below. Quantitative and qualitative results and triangulation of results are also presented. Only the quantitative results are followed by percentages and no correlation calculations were done. Additionally, combined results are presented following triangulation of data.

### 3.1. Human assets

Having their independence at home was found to be an enabler of older adults’ adaptation to extreme temperatures as they could do what they wanted to keep cool or warm during extreme hot and extreme cold temperatures, respectively (Table 3).

**Table 3:** Themes within each category of assets.

Human assets	Financial assets	Physical assets	Place-based assets	Social assets
Independence and control;	Managing competing expenses and still struggling;	Lack of insulation;	Indoor versus outdoor spaces;	I’m connected… to my family;
Return to the nest;	Savings should be savings;	Lacking cooling and heating devices;	Work the land;	I feel supported but I never ask for help;
Illiteracy and health illiteracy;	Thrifty and proud.		Ward level activities;	I socialise but not as much as I should.
Chronic illness not frailty.			Heatwave/Cold Weather Plan, what Plan?	

Additionally, most participants feared becoming frail and losing their physical and mental abilities (88.5%), which were found to constitute limitations to adaptation to extreme temperatures. These fears were most pronounced in relation to the risk of falls during extreme cold temperatures.

Changing relationships within the household due to unemployment or divorce had forced some of participants’ adult children to return home with impact on their economic and motivational capabilities to adapt.

Participants with the lowest literacy (primary school education or less: 57.7%) and lowest health literacy (hot/cold weather does not disrupt everyday life: 61.5%/67.4%; not received information/advice on what to do during hot/cold weather: 50.0%/54.3%) perceived more obstacles to action as they felt low literacy impacted on their ability to know what to do during extreme temperatures. Additionally, participants suggested that personalised advice provided by specialists (i.e. GP, Nurse, Community or Council officers) to fulfil personal needs would be preferable to general advice as given currently (e.g. Heatwave and Cold Weather Plans).

Participants with low perceived health status (poor or fair: 63.4%) mentioned it as a limitation to adaptation to extreme temperatures as it impacted on their perceived ability and motivation to act:

*‘Yes*, *now it’s worse maybe because of my illness (diabetes) […] I get too tired and it’s very hot.’ (VF[76])*

*‘Now I tolerate less very hot weather […] feel tired and don’t want to do anything*.*’ (MM[85])’*

Nevertheless, participants did not feel frail or vulnerable despite their age and health status, and highly valued their independence, self-care and endurance in adapting to extreme temperatures. These findings may help explain why older adults tend not to perceive extreme temperatures as a threat and as a result do not change their behaviours and actions during extreme temperature events.

### 3.2. Financial assets

Participants with low pensions (≤ 500 €/month) (46.1%) revealed that managing their income with fixed expenses (i.e. rent/mortgage, electricity, gas, water, food and medications) impacted on their health and well-being, and constituted a barrier in adapting to extreme temperatures. This included, for example, having to choose between a balanced nutrition or buying medication, and using cooling or heating devices during extreme temperatures (Table 3). This theme revealed that keeping warm during extreme cold temperatures put more pressure on their tight budget than keeping cool during hot temperatures, as they found it more difficult to keep their home warm during cold temperatures. As a result, participants’ income and perceived financial situation was decisive on how they prioritised and felt motivated to engage in cooling or warming behaviours.

Savings were found to be an enabler to action as a resource some participants used to pay for unanticipated expenses (e.g. housing or health), but not so much to be used to be able to better deal with extreme temperatures. For some, expenses such as fitting air conditioning or central heating in their homes could be afforded, but most chose not to due to the prospect of having to use savings to go to a good care home:

*‘If it’s too cold I turn on the heater*, *if it’s not I don’t. I go to bed early, I wear more layers of clothes. That’s all I do, I don’t do anything special.’ (AF[79])*

*‘If I am cold I put on one extra jumper or coat*, *nothing special’ (BBF[74])*

Careful budgeting and being thrifty constituted some of the qualities older adults found essential in dealing with their financial difficulties, enabling them to consider heating and cooling their homes during extreme temperatures. Despite this, they also realised that some of the choices made had negative impacts on their health and well-being. Being reluctant to use cooling and heating devices was one of them. Notwithstanding, those being able and wanting to use such devices did it only in one room of the house to save money, but the majority would not even consider that.

### 3.3. Physical assets

Most participants lived in old buildings (50 years or older) (69.3%) and in apartments (76.9%) with a lack of insulation (e.g. single-glazed windows, no wall and/or roof insulation), as well as a lack of cooling (e.g. electric fan, air conditioning) and heating devices (e.g. electric heater) (Table 3). These were considered obstacles to their ability to keep their homes and themselves cool and/or warm during extreme hot and/or cold temperatures, respectively. Tenants found it more difficult to keep cool and warm than homeowners, as the former were dependent on landlords’ decisions to invest in the property, whilst the latter could invest in improving housing quality. Despite this, indoor temperature was found to be a pervasive problem amongst participants in this study.

Furthermore, participants found cooling and heating the home to be a ‘waste’ of money due to insulation problems. Nevertheless, participants that perceived the health benefits of thermal comfort were willing to invest in using cooling and heating devices:

*‘I bought a fan (laughs), bought a fan with two blades*. *There was nothing else… (laughs).’ (IM[76])*

*‘If it’s too cold I turn on the heater*, *if it’s not I don’t.’ (AF[79])*

*‘If it’s very cold*, *very very cold I stay home […]. I stay more at home, I don’t like it but I do it.’ (AAF[75])*

### 3.4. Place-based assets

Lack of access to amenities such as social and cultural centres (60.8%) and public facilities (42.3%) were found to hinder older adults from keeping cool and warm during extreme temperatures. Additionally, mobility problems, lack of public transport and not having anyone to accompany them, were also mentioned as obstacles and constituted some of the reasons mentioned for not accessing local infrastructures and amenities (Table 3). Strategies used by some participants to keep cool or warm included going to shopping centres, supermarkets and coffee shops, and attending local and community centres, as these places would be acclimatised. Staying in their own gardens and going to public parks were also highlighted as ways to keep cool:

*‘When it’s very hot*, *I go to the supermarket or shopping centre. It’s cool there, there’s air conditioning.’ (AM[65])*

Despite this, the majority of participants revealed staying inside their homes during extreme temperatures. As most participants were born in the countryside and used to work in agriculture in their youth before moving to the city, they would keep themselves occupied and fit if they had access to land and could cultivate food. Despite this, working the land during extreme temperatures was mentioned as a health risk but did not dissuade them from doing it.

Alternatives to staying home during extreme temperatures included going to the countryside or beach. This was mainly possible to participants as some Lisbon wards organised days out during the summer months (June and September).

Awareness of the Heatwave and Cold Weather Plans was low among participants, but was found to be an enabler for action for those who were aware of such plans. One of the reasons mentioned for not being aware of such preparedness plans was due to the fact that they did not perceive extreme temperatures as a threat. Furthermore, past experiences were found to be the bedrock for future behaviours when dealing with extreme temperatures.

### 3.5. Social assets

Of married participants (28.8%), some felt they had to compromise due to their spouse’s preferences for cooling and/or heating.

Participants that had direct face to face contact at least once a week or more with their children (63.5%), extended family (11.5%), and friends and neighbours (88.4%) felt more connected and supported than those that had less frequent contacts (Table 3). Bonding social capital (e.g. children and neighbours) was found to be extremely important during extreme temperature events as participants would check and be checked by such contacts:

*‘My daughter checks on me everyday*. *She calls and comes to my house to see how I am’ (JF[83])*

Additionally, bridging social capital was also found to play an important role as participants shared advice with old acquaintances on how to best respond to extreme temperatures. Despite this, even those that felt connected and supported revealed never or almost never asking others for help. As a result, linking social capital provided by staff and officials from community organisations and local council were a vital lifeline for the most isolated participants. Social capital was thus found to be an enabler for feeling supported and confident in responding to extreme temperatures.

Family and friends were the most important sources of instrumental, emotional and informational social support to participants, and family was considered to be the most trustworthy source of support. Nevertheless, if faced with financial difficulties (financial assets) participants would not consider asking for financial support from anyone. This was found to be an obstacle to being able to keep cool and warm in their homes. Participants favoured receiving individualised advice that considered their personal health and well-being, and not just mass media information. The lack of social activities in their local area were found to be reasons why older adults would stay more time at home and alone.

### 3.6. Managing the assets portfolio

The ability of participants to adapt to extreme temperatures was found to be dependent on asset context and diversity, and the dynamics through which extreme temperatures enhanced or reduced the stock of assets (tangible and intangible) available to older adults. A summary of the themes exploring the role of assets in older adults’ adaptation to extreme temperatures is shown in Table 3.

The life circumstances of participants in this study have changed dramatically throughout the years both due to intrinsic (e.g. age, health status) and extrinsic factors (e.g. economic and social changes).

Overall, participants experienced a variety of circumstances which influenced the accessibility to and availability of different types of assets that impacted on their adaptation to extreme temperatures. As a result, and in order to overcome such difficulties, participants engaged in activities of assets replacement, exchange or substitutions by which the existence of one type of asset could compensate for the inexistence of another type of asset. Examples included the substitution of income/pensions (financial assets) to improve insulation, buy cooling or heating devices (physical assets) and pay energy bills to maintain thermal comfort. Despite this, the substitution or replacement of assets was not frequently mentioned by the majority of participants, most of them instead preferring to be thrifty.

### 3.7. Adaptation to extreme heat and extreme cold–(dis)similarities

The findings above have highlighted the diversity of assets and adaptive strategies used by older adults. Despite this, many participants recognised many constraints and limits to their ability to adapt and protect their health and well-being from the risks and impacts of extreme temperatures (Table 4). This was found to be due to the lack of access and availability of assets.

**Table 4:** Constraints and limits to adaptation drawing on assets.

Category of assets	Extreme Heat	Extreme Cold
**Human assets**		
Health beliefs or misconceptions	Cooling is bad for health; Fan and air conditioning is bad for health.	Heating is bad for health; Heating only one room of the house is worse than no heating; Fear of flu vaccine
Lack of information, knowledge and education	on the best adaptation options to implement.	on the best adaptation options to implement.
**Financial assets**		
Expenses	High electricity prices	High electricity and gas prices
Pensions	Low income; Austerity measures; Cuts in pensions; Cooling not a priority.	Low income; Austerity measures; Cuts in pensions; Heating not a priority.
**Physical assets**		
Technological	Not owning cooling devices	Not owning heating devices
Technological beliefs	Not liking cooling devices; Fear of poor air quality and disease by using air conditioning (e.g. changing filters)	Not liking heating devices; Fear of gas or electrical due to old house and old electrical installations
Built environment	Lack of insulation; Hot house; Building codes; Low quality housing stock;; Single-glazed windows; Living on the top or ground floors; Lack of safety	Lack of insulation; Cold and damp house; Low quality housing stock; Single-glazed windows; Living on the top or ground floors; Lack of safety; Fear of falls
Housing tenure	Renting; No refurbishments; Inability to move house (both tenants and owners)	Renting; No refurbishments; Inability to move house (both tenants and owners)
**Place-based assets**		
Early Warning Systems	Lack of awareness and knowledge	Lack of awareness and knowledge
Urban planning and Green spaces	Lack of trees and shade	Fear of falls
**Social assets**		
Social contacts	Lack of company to go to places; Isolation; Reluctant to ask for help	Isolation; Reluctant to ask for help
Social activities and participation	Isolation; Lack of sense of community	Isolation; Lack of sense of community

Feeling that using cooling and heating devices is harmful for health (human asset) due to the air movement they cause (e.g. fan, air conditioning) and only being able to afford heating one room which was considered to be worse for health than not using any heating (human and financial assets) were reasons mentioned for not using such devices. Lack of information and misinformation about safety of the devices (i.e. gas, electrical) and air quality were also mentioned as constraints. Therefore, participants would like information on the best adaptation strategies for health to be available to everyone, especially to those with certain health conditions.

Electricity and gas prices, and as a result, cooling and heating costs (financial assets) also restrained older adults from being able to adapt due to more pressing priorities such as paying the rent, medication and food. Participants were reluctant and thrifty preferring to save any money for other pressing needs (financial asset), and owning and using heating and cooling (physical asset) were not amongst these.

Housing quality and insulation problems (physical asset) were also found to be reasons not to own or use heating or cooling devices. As most participants lived in rented accommodation, landlords were found to not be held accountable for their responsibility to improve and ensure the quality of their homes. Despite this, participants were not able to move house due to low pensions (financial asset).

The use of public parks and gardens during extreme heat was limited due to safety issues and lack of shade and trees (place-based asset). Distance to green spaces was also found to limit their ability to keep cool. On the other hand, the use of public indoor spaces (place-based asset) during extreme cold was not common as participants feared falling as cold restricted their movements and ability to walk on pavements as they become very slippery. Awareness of the Heatwave and Cold Weather Plans (place-based assets) was also found to be low which was due to a lack of interest in being informed and lack of awareness of the health risks, as well as not feeling vulnerable to extreme temperatures.

As a result of some of the findings mentioned above, most participants tended to spend most of their time at home and alone, which contributed to low levels of social participation and reduced social activities and support (social assets). These findings show clear similarities between the constraints and limits participants faced in adapting to extreme heat and cold temperatures.

### 3.8. Opportunities for enhancing older adults’ adaptation to extreme temperatures

Following on the findings from previous sections, participants were also asked if there was anything that could improve their adaptive strategies during extreme temperatures. This allowed older adults to express what they felt could help them support and enhance their adaptation. Nevertheless, many participants (61.0%) struggled to express their opinions and some even declined from both realistically and hypothetically revealing what could be improved in their lives to allow for better adaptation strategies. Participants unravelled some of the deeper roots of such powerlessness and helplessness which also revealed passiveness in their ways of life. Feelings that no one does anything for them or gives anything to them, that they can only rely on themselves, that it is a ‘save yourself if you can’ world, revealed lack of social capital with a great reliance on individual and household actions and reduced or none collective action and state or private actors action. Moreover, these participants were acting almost as if there was no solution for improving adaptation. They felt that there was no reason in pointing that out because they know they cannot do anything about it and that no one will do anything about it either. This was found to be in itself a constraint and limit for action for enhancing adaptation.

Despite this, some participants (39.0%) described possible ways in which opportunities building on assets could improve their adaptation to extreme temperatures (Table 5). These ranged across all types of assets, both tangible and intangible, and included better education and individualised information and advice (human assets); incentives or subsidies to reduce adaptation costs and increased/additional income (financial assets); building and home refurbishments for better insulation and energy-efficiency, access to heating and cooling technologies (physical assets); improved public infrastructures, spaces and services, access to social activities (place-based assets).

**Table 5:** Adaptation opportunities suggested by participants to improve their adaptation to extreme temperatures.

Category of assets	Extreme Heat: examples	Extreme Cold: examples
**Human assets**		
Educational	Life-long education; Knowledge sharing and learning; Communication through media.	Life-long education.
Informational	Individualized advice by health professionals.	Individualized advice by health professionals.
**Financial assets**		
Incentives and subsidies (e.g. fund, grant)	Financial incentives to improve insulation, install air conditioning. Electricity subsidies to reduce costs.	Financial incentives to improve insulation, install heating devices. Gas and electricity subsidies to reduce costs.
Income	Increased pensions. Reduce austerity measures. Seasonal migration.	Increased pensions. Reduce austerity measures.
**Physical assets**		
Housing	Better housing. Well insulated home. Adapted homes. Enforce responsibilities and liabilities of landlords to refurbish/repair old homes.	Better housing. Well insulated home. Enforce responsibilities and liabilities of landlords to refurbish/repair old homes.
Urban planning laws and regulations	Enforce building standards. Alter building codes to allow installation of air conditioning.	Enforce building standards.
Engineered and built environment	Enforce building codes; Improve insulation.	Enforce building codes; Improve insulation.
Technological	Air conditioning.	Central heating.
**Place-based assets**		
Green infrastructure / Aforestation	Create shade; Improve quality and safety of gardens and parks.	-
Services	Social safety nets; Social protection; Food banks and distribution of food surplus; Free healthcare and transports.	Free healthcare and transports.
Policies and Programs	Health Early Warning Systems; City-level plans; Local action to support older people.	Local action to support older people. Help in managing personal budget; Home care; Befriending programs.
**Social assets**		
Activities	Local activities for older people, even during summer months.	Local activities for older people.
Participation	Develop old people and intergenerational participation in society.	Develop old people and intergenerational participation in society.

Adapting to both extreme heat and cold temperatures constituted a challenge for participants in this study. Despite this, adaptation to extreme cold was found to be more challenging than adaptation to extreme heat. Health status (e.g. chronic disease) and perceptions of inability to effectively adapt through the options available to them (i.e. assets) were the most important reasons mentioned. Participants felt unable to do more than what they were already doing, despite it not being enough to keep them cool or warm during extreme heat or cold, respectively (i.e. limits to adaptation). Therefore, most adaptations implemented by participants were non-technological and did not include the use of electrical devices (e.g. fan, air conditioning, heater). The use of technological adaptations was limited as a result of not owning such devices and cost related to using them. Constraints and limits to adaptation were associated with health beliefs, safety of gas and electric devices, gas and electricity prices, as well as housing insulation deficiencies. Access to and availability of both tangible (financial, physical and place-based) and intangible assets (human and social) determined when and how older adults engaged in adaptation behaviours.

## 4. Discussion

In this study, participants’ asset portfolio were found to determine their ability to adapt to extreme temperatures. Extreme temperatures were found to increase pressure on existing human assets (e.g. health status) with implications for the way older adults responded to extreme temperatures. Other studies in the United States of America (USA), France and internationally [1, 4, 52] also found that the lack of mobility had adverse consequences on older adults’ ability to respond to extreme heat. Additionally, the fear of falls was frequently mentioned in a study in the United Kingdom (UK) as a threat to becoming incapacitated especially during extreme cold [53] due to thermally inefficient housing and low temperatures. The detrimental effects of chronic diseases (e.g. cardiovascular and respiratory diseases) in the ability to respond to extreme cold temperatures have also been emphasized [54]. The results of this study are also comparable to those in Western Europe, in the UK and internationally [55–57] who found that low educational levels impact and constitute a barrier to older adults’ ability to respond to extreme heat.

Reduced pensions, pride in not asking for help, struggling alone to afford essential housing expenses such as cooling and heating (i.e. financial assets) also acted as constraints and influenced how participants adapted to extreme temperatures. Participants prioritised using their financial assets to buy food and medication, but were aware that such choices had impacts on their health and well-being. The results of this study are comparable to those in the USA where participants only used cooling devices (e.g. air conditioning) if they felt able to afford them [58]. Feeling hot or cold at home was a ‘normal’ feature during extreme heat and cold temperatures, respectively. Extreme temperatures were found to pose great pressure on already limited financial assets. Low income was thus found to be the greatest constraint contributing to inability to adapt to extreme temperatures both in Europe and New Zealand [53, 59, 60]. Addressing issues such as electricity and gas prices, as well as subsidies for older adults living with low pensions were considered to be essential opportunities in reducing the pressure on financial assets and would act as facilitators for adaptation.

Lack of physical assets such as housing insulation, cooling and heating devices was the reality participants faced. The findings of this study suggest that poor housing conditions contribute as constraints to the ability to effectively adapt to extreme temperatures [1, 57]. Landlords’ underinvestment in insulation constituted one of the biggest constraints and limitations for adapting to extreme temperatures in this study as most participants lived in rented accommodation.

Even if place-based assets (e.g. green spaces, shopping centres) were available to keep cool or warm outside their homes not many participants used them. However, they contrast with the findings from France and the UK, where the lack of green spaces was found to reduce the ability to adapt to extreme hot temperatures [1, 57].

Weak social networks (social assets) were also found to be limits to action and have negative impacts in accessing built infrastructure, as was also found in an UK study [61]. Additionally, knowledge of the Heatwave and Cold Weather Plans was also reduced. As a result, information on the availability of and access to public spaces could according to participants in this study constitute an opportunity as well as an alternative to stay in a hot or cold house during extreme heat and cold temperatures, respectively. These study’s findings agree with those of research undertaken in the UK where older adults were mostly supported by their close family and neighbours during extreme temperatures [62]. Extreme heat posed a greater threat to social isolation as participants took refuge in their homes, but they would still go out during extreme cold events. The findings of this study also support those in the USA and UK where social isolation, lack of social capital and networks (social assets) are found to be constraints and contribute to increased inability to adapt to extreme temperatures [52, 57] with impacts on access to other assets such as financial and physical assets. Social activities and having a community centre were enablers of social assets and helped participants keeping cool or warm as these places had cooling and heating devices, respectively allowing thermal comfort (physical assets).

Overall, participants in this study did not see themselves as old or frail and only a minority perceived themselves at risk from extreme temperatures, despite fear of mental and physical risks such as dementia and falls. Such participants valued their independence, self-care and endurance. Additionally, the fear of falls in the home and outdoors, and becoming incapacitated as a result were mentioned in relation to extreme cold temperatures. Similar findings were obtained in Australia [15–17] and the UK [11–13] which may lead older adults to be at greater risk of health impacts.

Participants revealed that there are a range of opportunities for enhancing their adaptation strategies drawing on assets that they would welcome. The findings of this research provide a range of contributions to policy and practice for reducing the human health impacts of extreme temperatures, such as increasing low pensions, reducing energy costs and improving social networks. This study indicates these can be achieved through the planning, development and implementation of policies and actions aiming at improving adaptation. In order to accomplish this, a core focus on increasing assets, both access and availability, as well as quality and quantity of each type of assets and overall asset portfolio is key. To increase all types of assets requires sufficient funding and political commitment for the short-, medium- and longer-term and an investment in tailored national and local policy decisions and interventions. Additionally, Portugal still needs to develop and enhance communication of policies and procedures between government agencies and citizens. Focusing on assets will require a shift in the passive way in which older people living independently in Lisbon, Portugal are currently informed and advised or made aware of the Heatwave and Cold Weather Plans, with no further actions being put in place to ensure they do not suffer the health impacts of extreme temperatures. As a result, new ways in which information is distributed needs to be carefully considered and adjusted to different types of people in order to allow an easy and equitable access to advice. Special attention should also be given to the type of adaptation measures provided in such advice so they are non-exclusionary and suitable to older people’s daily lives and rhythms.

All in all, the government, its departments, bodies and public bodies, public health and social care authorities should work together with other organizations and institutions including community and voluntary organizations to develop feasible priorities and ensure that an integrated and people-centred approach is put in place for the benefit of older people. This research indicates that local and community organizations and institutions would be more appropriate to implement such measures, as they are closer and more accessible to older people. These organizations and institutions could aim at working together in examining particular aspects and characteristics of older people’s lives that are crucial to respond to temperature extremes and implement measures, to: improve health status; strengthen the capacity of the individual to know what to do in the case of extreme temperatures and to be proactive; improve the general awareness of risks and impacts by the individual; improve social networks around the individual, and strengthen the links between health and social care teams; reducing the cost of going to the GP compared with overall income; implement policy measures to reduce poverty, hunger and improve the quality of the housing stock. Ensuring a focus on assets and in increasing older people’s agency and empowerment are also essential for better adaptation.

Unlike previous studies that have narrowly focused on disciplinary perspectives, a single extreme temperature event (heat or cold) and a restricted range of specific factors, the approach taken here deliberately sought to respond to theoretical and operational imperatives for better understanding what shapes adaptation.

In summary, this study extends existing research by taking an integrated approach in investigating access to and availability of all categories of assets, both tangible assets (financial, physical and place-based) and intangible assets (human and social), rather than in isolation as it has been done in previous research. It showed that the vast majority of older adults face restrictions in availability and access to assets with impacts on how they adapt to extreme temperatures. This study has also revealed the key role assets play in shaping older adults’ ability to respond to extreme temperatures. It has also made a significant theoretical contribution to the assets and asset-based literatures as the concept of assets allows a focus on positive characteristics and capacities of individuals. Overall, it highlights the need for asset-based approaches and interventions to understanding and promoting health and wellbeing, and prevent adverse health effects of extreme temperatures amongst older adults.

Opportunities for improving older adults’ adaptation were found to exist, disentangling areas where strategies, action and investment can be achieved through a health in all policies (HiAP) approach. These areas constitute feasible options to be considered using an integrating framework at the local and national levels using a sustainable development lens for achieving the Sustainable Development Goal (SDG) 3 –Good Health and Well-Being through its interactions with other SDGs (e.g. SDG1—No Poverty; SDG4 –Quality Education; SDG7 –Affordable and Clean Energy; SDG10 –Reduced Inequalities; SDG11 –Sustainable Cities and Communities) (see [63]).

Supporting older adults’ adaptation to extreme temperatures is possible and participants in this study have revealed how it is possible. These efforts can in addition be complemented with efforts to reduce older adults’ vulnerability and building resilience to extreme temperatures.

The research carried out here and the findings obtained highlight prospects for further research focusing on understanding how individual vulnerability and resilience to extreme temperatures are shaped in order to allow better adaptation, as well as the links with the concept of assets and sustainable development.

## Supporting information

S1 Table Descriptive quantitative results for specific instances of the five asset types. (DOCX)

S1 File Phase 1 and 2 interview protocols: Original Portuguese Text. (DOCX)

S2 File Phase 1 and 2 interview protocols: English Translation of Original Portuguese Text. (DOCX)
