# Supplementary material for: The contribution of assets to adaptation to extreme temperatures among older adults
Source: PLoS One. 2018 Nov 29;13(11):e0208121. doi: 10.1371/journal.pone.0208121 (PMC6264854; doi:10.1371/journal.pone.0208121)
Supplement: S1 Table — (DOCX) [file pone.0208121.s001.docx]

**Descriptive quantitative results for specific instances of the five asset types**

|  |  | Percentages (%) |
| --- | --- | --- |
| **Human assets** | Percentage of individuals living alone | 57.7 |
|  | Percentage of individuals that have no formal education | 17.3 |
|  | Percentage of individuals in lower supervisory and technical occupations, semi-routine and routine occupations, or never worked | 71.1 |
|  | Percentage of individuals with poor health | 19.2 |
|  | Percentage of individuals with current health worse than a year ago | 30.8 |
|  | Percentage of individuals hampered in their daily activities | 48.1 |
| **Financial assets** | Percentage of individuals that have financial difficulties | 42.4 |
|  | Percentage of individuals with monthly income ≤500 euros | 46.1 |
|  | Percentage of individuals that have pensions as source of income | 94.2 |
|  | Percentage of individuals that have difficulties paying the housing expenses | 28.8 |
|  | Percentage of individuals that have difficulties paying for food | 26.9 |
|  | Percentage of individuals that have difficulties paying for healthcare or medication | 30.8 |
| **Physical assets** | Percentage of individuals that live in apartment buildings | 76.9 |
|  | Percentage of individuals that live on the first floor or above | 53.8 |
|  | Percentage of individuals that do not have lift in the building | 82.7 |
|  | Percentage of individuals that live in houses with 50 years or older | 69.3 |
|  | Percentage of individuals that are not satisfied with their house | 11.5 |
|  | Percentage of individuals that live in rented homes or social housing | 61.5 |
|  | Percentage of individuals that are not happy with their living conditions | 59.6 |
|  | Percentage of individuals that do not own a landline phone | 13.5 |
|  | Percentage of individuals that do not own a mobile phone | 15.4 |
|  | Percentage of individuals that do not own a TV | 0.0 |
|  | Percentage of individuals that do not own a radio | 3.8 |
|  | Percentage of individuals that do not own a computer | 65.4 |
|  | Percentage of individuals that do not own a car | 69.2 |
| **Place-based assets** | Percentage of individuals that do not have a food store or supermarket within walking distance | 11.5 |
|  | Percentage of individuals that do not have a post office within walking distance | 50 |
|  | Percentage of individuals that do not have banking facilities within walking distance | 32.7 |
|  | Percentage of individuals that do not have a cinema, theatre or cultural centre within walking distance | 60.8 |
|  | Percentage of individuals that do not have public transport facilities within walking distance | 0.0 |
|  | Percentage of individuals that do not have access to private and public spaces close to their house, where they can sit and relax, have a coffee, or talk peacefully to neighbours, acquaintances and friends | 13.5 |
|  | Percentage of individuals that do not have access to public facilities close to their house where they can practice physical activity (such as playgrounds, parks, sport centres, swimming pools, etc.) | 42.3 |
|  | Percentage of individuals that rate their neighbourhood as bad or very bad | 15.4 |
|  | Percentage of individuals that rate health services as bad or very bad | 26.9 |
|  | Percentage of individuals that rate public transport as bad or very bad | 35.3 |
|  | Percentage of individuals that rate care services for the elderly as bad or very bad | 26.4 |
|  | Percentage of individuals that rate state pension system as bad or very bad | 64.7 |
|  | Percentage of individuals that do not go to private and public spaces | 50.0 |
|  | Percentage of individuals that do not go to public facilities for physical activities | 71.2 |
| **Social assets** | Percentage of individuals that do not have people available who can help them when they need | 25.5 |
|  | Percentage of individuals that do not have people that care for them | 2.0 |
|  | Percentage of individuals that have direct social contact with any of their children once or twice a month or less | 36.5 |
|  | Percentage of individuals that have direct social contact with any brother, sister or other relative once or twice a month or less | 88.5 |
|  | Percentage of individuals that have direct social contact with any friends or neighbours once or twice a month or less | 11.5 |
|  | Percentage of individuals that have indirect social contact with any of their children once or twice a month or less | 5.7 |
|  | Percentage of individuals that have indirect social contact with any brother. sister or other relative once or twice a month or less | 48.1 |
|  | Percentage of individuals that have indirect social contact with any friends or neighbours once or twice a month or less | 54.9 |
|  | Percentage of individuals that take part in social activities less or much less than most people their age | 36.5 |
|  | Percentage of individuals that take part in caring for and educating children less often than once a week | 82.7 |
|  | Percentage of individuals that take part in cooking and housework less often than once a week | 11.6 |
|  | Percentage of individuals that take part in caring for elderly/disabled relatives at least once a week | 5.8 |
|  | Percentage of individuals that take part in voluntary and charitable activities less often than once a week | 92.3 |
|  | Percentage of individuals that take part in activities organized in their local area less often than once a week | 23.1 |
|  | Percentage of individuals that spend too little time in contact with family members | 60 |
|  | Percentage of individuals that spend too little time in other social contact (not family) | 44.2 |
|  | Percentage of individuals that spend too little time in their own hobbies/interests | 32.7 |
|  | Percentage of individuals that spend too little time taking part in voluntary work or political activities | 78.8 |
|  | Percentage of individuals that would find it difficult or very difficult to borrow money. if in serious financial difficulties | 60 |
