# Supplementary material for: The contribution of assets to adaptation to extreme temperatures among older adults
Source: PLoS One. 2018 Nov 29;13(11):e0208121. doi: 10.1371/journal.pone.0208121 (PMC6264854; doi:10.1371/journal.pone.0208121)
Supplement: S1 File — (DOCX) [file pone.0208121.s002.docx]

# Phase 1 and 2 Interview Protocols: Original Portuguese Text

**PROTOCOLO DE ENTREVISTA GERAL ESTRUTURADA E DE CALOR SEMI-ESTRUTURADA**

**FASE 1 - GERAL E CALOR**

**SECÇÃO A – INFORMAÇÃO SÓCIO-DEMOGRÁFICA**

1. Anotar o sexo observado: Feminino (1), Masculino (2)

2. Qual é a sua idade? ____ (anos)

3. Qual é o seu estado civil actual?

| Casado(a) | Divorciado(a) | Viúvo(a) | Solteiro(a) |
| --- | --- | --- | --- |
| 4 | 3 | 2 | 1 |

4. Vive com ...?

| Esposo(a) | Outros familiares (filhos, irmãos, irmãs, etc) | Outros não-familiares (amigos, colegas) | Sozinho(a) |
| --- | --- | --- | --- |
| 4 | 3 | 2 | 1 |

5. Qual o nível de ensino mais elevado que completou?

| Sem nível de ensino | 1 |
| --- | --- |
| 1º Ciclo | 2 |
| 2º Ciclo | 3 |
| 3º Ciclo | 4 |
| Secundário | 5 |
| Bacharelato | 6 |
| Licenciatura | 7 |
| Mestrado | 8 |
| Doutoramento | 9 |
| Nenhum dos anteriores | 10 |
| Não sabe | 11 |

**SECÇÃO B – ESTADO DE SAÚDE,**

Agora vou perguntar-lhe sobre a sua saúde, bem-estar e a sua vida

6. Em geral, diria que a sua saúde é:

| Óptima | Muito boa | Boa | Razoável | Fraca |
| --- | --- | --- | --- | --- |
| 5 | 4 | 3 | 2 | 1 |

7. Comparando com o que acontecia há um ano, como descreve o seu estado geral actual:

| Muito melhor | Com algumas melhoras | Aproximadamente igual | Um pouco pior | Muito pior |
| --- | --- | --- | --- | --- |
| 5 | 4 | 3 | 2 | 1 |

8. Está de alguma forma limitado(a) nas suas actividades diárias devido a uma doença prolongada, uma deficiência, um problema de mobilidade, ou problema de saúde do foro psicológico?

| Sim, muito | Sim, de alguma forma | Não | NS/NR |
| --- | --- | --- | --- |
| 1 | 2 | 3 | 8 |

9.1. Se sim: Qual? _________________________________

**SECÇÃO C – INFORMAÇÃO sobre HABITAÇÃO e EQUIPAMENTOS**

**Apenas** **algumas perguntas sobre a** **sua casa e bairro**

9. Qual a opção que melhor descreve a sua casa?

| Moradia unifamiliar independente | Moradia multifamiliar ou duplex | Moradia em banda | Apartamento num prédio até 3 andares | Apartamento num prédio com 4 até 8 andares | Apartamento num prédio com 9 ou mais andares |
| --- | --- | --- | --- | --- | --- |
| 1 | 2 | 3 | 4 | 5 | 6 |

10. Em que andar está situada a sua casa? ______

11. Existe elevador no edifício?

| Não | Sim, e funciona | Sim, mas não funciona | NS/NR |
| --- | --- | --- | --- |
| 1 | 2 | 3 | 8 |

12. Qual a idade do edifício onde vive?

| Menos de 10 anos | 10-19 | 20-29 | 30-39 | 40-49 | 50-59 | 60-74 | 75-99 | 100 e mais | NS/NR |
| --- | --- | --- | --- | --- | --- | --- | --- | --- | --- |
| 1 | 2 | 3 | 4 | 5 | 6 | 7 | 8 | 9 |  |

13. Numa escala de 1 a 5, qual a satisfação com a sua casa?

| Muito insatisfeito |  |  |  | Muito satisfeito |
| --- | --- | --- | --- | --- |
| 1 | 2 | 3 | 4 | 5 |

14. A casa onde reside é …?

| Própria | Arrendada | Habitação Municipal |
| --- | --- | --- |
| 1 | 2 | 3 |

15. Está contente com as actuais condições da sua casa ou mudava de casa se tivesse possibilidade?

| Estou contente | Mudava |
| --- | --- |
| 1 | 2 |

22.1. Se mudava: Porque mudava?__________________________

16. Qual(is) dos seguintes equipamentos/bens possui? 16.7. Se sim: Usa-os?

|  | Sim | Não |  | Sim | Não |
| --- | --- | --- | --- | --- | --- |
| 16.1.Telefone fixo | 1 | 2 |  | 1 | 2 |
| 16.2. Telemóvel |  |  |  |  |  |
| 16.3. Televisão |  |  |  |  |  |
| 16.4. Rádio |  |  |  |  |  |
| 16.5. Computador |  |  |  |  |  |
| 16.6. Carro |  |  |  |  |  |

**SECÇÃO D – FINANÇAS/RENDIMENTOS**

17. Como descreveria a actual situação financeira da sua família?

| Muito confortável | Confortável | Temos de ter cuidado, mas conseguimos aguentar-nos | Temos dificuldade em chegar ao fim do mês com dinheiro | As coisas estão muito difíceis | NS/NR |
| --- | --- | --- | --- | --- | --- |
| 5 | 4 | 3 | 2 | 1 |  |

18. Por favor, pode dizer-me qual o rendimento médio mensal do seu agregado familiar (depois dos importos)? Se não souber o valor exacto, por favor diga uma estimativa.

| ≤ 350 euros | 351-500 | 504-800 | 801-1500 | 1501-2500 | > 2500 euros |
| --- | --- | --- | --- | --- | --- |
| 1 | 2 | 3 | 4 | 5 | 6 |

19. Nos últimos 12 meses o(a) Sr.(a) ou alguém do seu agregado recebeu algum dos seguintes tipos de rendimento?

|  | Sim | Não | NS/NR |
| --- | --- | --- | --- |
| 19.1. Uma remuneração ou um salário | 1 | 2 | 8 |
| 19.2. O rendimento de uma profissão liberal ou de uma exploração agrícola |  |  |  |
| 19.3. Uma pensão |  |  |  |
| 19.4. Um subsídio de desemprego, subsídio para deficientes ou outro benefício social |  |  |  |
| 19.5. Outro rendimento (ex: poupanças, propriedade, investimentos, etc.) |  |  |  |

20. Nos últimos 2 anos, teve dificuldades em pagar a totalidade das despesas da casa? (i.e. renda ou amortização da casa, contas de electricidade, água, gás)

| Não, nunca | Sim, às vezes | Sim, frequentemente | NS/NR |
| --- | --- | --- | --- |
| 3 | 2 | 1 | 8 |

Se sim: 20.1. Quais?__________ 20.2. Porquê?__________

21. Alguma vez durante os últimos 12 meses, houve dificuldades para comprar comida?

| Sim | Não | NS/NR |
| --- | --- | --- |
| 1 | 2 | 8 |

21.1. Se sim: Pode falar um pouco sobre isso?

22. Alguma vez durante os últimos 12 meses, houve dificuldades em pagar serviços de saúde ou medicamentos?

| Sim | Não | NS/NR |
| --- | --- | --- |
| 1 | 2 | 8 |

22.1. Se sim: Pode falar um pouco sobre isso?

23. Se, por alguma razão, tivesse sérias dificuldades financeiras e tivesse de pedir dinheiro emprestado para conseguir viver, quão difícil ou fácil acha que isso seria:

| Muito difícil | Difícil | Nem fácil nem difícil | Fácil | Muito fácil | NS/NR |
| --- | --- | --- | --- | --- | --- |
| 1 | 2 | 3 | 4 | 5 | 8 |

23.1. Porquê?_______________________________________________________________________

23.2. A quem pediria dinheiro? *(anotar relação com o participante)* __________________________

**INFORMACAO SOBRE CALOR**

**SECÇÃO E – EXPERIÊNCIAS DE CALOR, PRÁTICAS QUOTIDIANAS E RESPOSTAS AO CALOR**

Agora vou fazer-lhe algumas perguntas sobre como se sente e o que faz quando o tempo está muito quente. Vou também fazer-lhe algumas perguntas sobre a sua casa e sua saúde durante o tempo muito quente.

24. Quando foi a última vez que esteve muito calor nos últimos anos?

25. Onde estava nesse momento?

26. Houve alguma coisa que tenha começado a fazer para se proteger do tempo muito quente?

| Sim | Não | NS/NR |
| --- | --- | --- |
| 1 | 2 | 8 |

26.1. Se sim: O que começou a fazer? Em sua casa? Na rua? (Coisas relacionadas com a sua casa? Coisas relacionadas com o que veste? Coisas relacionadas com o que come?)

27. Houve alguma coisa que quisesse fazer mas não podesse fazer?

| Sim | Não | NS/NR |
| --- | --- | --- |
| 1 | 2 | 8 |

45.1. Se sim: O quê?

Porque é que não pôde fazer?

28. Quando está muito calor, como se mantém fresco(a) em sua casa durante dia?

O que faz?

Onde vai?

29. E durante a noite?

O que faz?

Onde vai?

30. Quando está muito calor, como se mantém fresco(a) na rua?

O que faz?

Onde vai?

31. Acha que é mais afectado(a) pelo tempo muito quente que outras pessoas?

| Sim | Não | NS/NR |
| --- | --- | --- |
| 1 | 2 | 8 |

31.1. Se sim: Pode dizer-me porquê? De que maneira?

32. Consegue pensar em quaisquer outros grupos de pessoas que podem ser mais afectados pelo tempo muito quente?

| Sim | Não | NS/NR |
| --- | --- | --- |
| 1 | 2 | 8 |

32.1. Se sim: Em quem está a pensar?

Pode dizer-me porquê?

33. Consegue pensar em algumas formas que o tempo muito quente pode afectar a saúde das pessoas?

| Sim | Não | NS/NR |
| --- | --- | --- |
| 1 | 2 | 8 |

33.1. Se sim: Pode falar um pouco mais sobre isso?

34. O tempo muito quente impede-o(a) de fazer coisas que costuma fazer na sua vida diária?

| Sim | Não | NS/NR |
| --- | --- | --- |
| 1 | 2 | 8 |

34.1. Se sim: Porquê?

Quais as coisas que gostaria de fazer, mas não pode por causa do tempo muito quente?

**SECTION F– INFORMAÇÃO**

35. Recebeu alguma informação ou conselho sobre o que fazer durante o tempo muito quente?

| Sim | Não | NS/NR |
| --- | --- | --- |
| 1 | 2 | 8 |

35.1. Se sim: Foi você que pediu essa informação ou conselho?

| Sim | Não | NS/NR |
| --- | --- | --- |
| 1 | 2 | 8 |

35.1.1. Se sim: A1) A quem pediu informação ou conselho?
              A2) O que lhe disseram?
                A3) Quando / Onde?
                  A4) Foi útil? De que maneir
           35.1.2. Se não: B1) Quem lhe deu a informação ou o conselho?
             B2) O que lhe disseram?
                                        B3) Quando / Onde?
                                        B4) Por que acha que lhe foi dada essa informação ou conselho?
                                        B5) Foi útil? De que maneira?

36. Forneceu alguma informação ou conselho a alguém sobre o que fazer durante o tempo muito quente?

| Sim | Não | NS/NR |
| --- | --- | --- |
| 1 | 2 | 8 |

36.1. Se sim: A quem?

O que disse?

37. Quanta informação acha que tem?

| Muita | Alguma | Pouca | NS/NR |
| --- | --- | --- | --- |
| 3 | 2 | 1 | 8 |

38. Gostaria de ter (mais) informação ou conselhos sobre o que fazer durante o tempo muito quente?

| Sim | Não | NS/NR |
| --- | --- | --- |
| 1 | 2 | 8 |

38.1. Se sim: Que fonte(s) de informação preferiria?

| Contactos sociais (família, amigos, vizinhos) | Professional de saúde | Rádio | TV | Jornal | Internet | Outra | NS/NR |
| --- | --- | --- | --- | --- | --- | --- | --- |
| 1 | 2 | 3 | 4 | 5 | 6 | 7 | 8 |

38.2. Porquê?

39. Acha que o tempo está a ficar mais quente de ano para ano?

| Sim | Não | NS/NR |
| --- | --- | --- |
| 1 | 2 | 8 |

39.1. Se sim: Pode falar um pouco mais sobre isso?

**SECTION G – CONTACTOS SOCIAIS E SAÚDE DURANTE O TEMPO MUITO QUENTE**

**Agora vou** **perguntar-lhe sobre** a **sua família e amigos durante o tempo muito quente.**

40. Com que frequência tem contacto com outras pessoas durante o tempo muito quente?

| Mais do que uma vez por dia | Todos os dias ou quase todos os dias | Pelo menos uma vez por semana | Uma ou duas vezes por mês | Menos frequentemente | NS/NR |
| --- | --- | --- | --- | --- | --- |
| 5 | 4 | 3 | 2 | 1 | 8 |

41. Quando está preocupado ou necessita de ajuda durante o tempo muito quente, a quem pede ajuda ou em quem pode confiar? *[anotar a relação com o participante]*

**Agora vou** **perguntar-lhe sobre** a **sua saúde durante o tempo muito quente**.

42. A sua saúde física limita o que pode fazer durante o tempo muito quente?

| Sim | Não | NS/NR |
| --- | --- | --- |
| 1 | 2 | 8 |

42.1 Se sim: Porquê?

De que forma?

43. O que faria se se sentisse mal/doente durante o tempo muito quente? *(Mais alguma coisa?)*

**SECTION H – CARACTERÍSTICAS DA RESIDÊNCIA**

**Apenas algumas perguntas** **sobre a sua casa** **durante o verão**

44. Considera que a temperatura dentro de sua casa no Verão é um problema?

| Sim | Não | NS/NR |
| --- | --- | --- |
| 1 | 2 | 8 |

45. Consegue manter-se fresco(a) em sua casa durante o verão?

| Sim | Não | NS/NR |
| --- | --- | --- |
| 1 | 2 | 8 |

45.1. Se sim: Como?

45.2. Se não: Porque não?

46. Consegue manter a sua casa fresca durante o verão?

| Sim | Não | NS/NR |
| --- | --- | --- |
| 1 | 2 | 8 |

46.1. Se sim: Como mantém a sua casa fresca?

46.2. Se não: 46.2.1. Porque não?

46.2.2. Acha que sentir calor em sua casa tem efeitos na sua vida em geral?

| Sim | Não | NS/NR |
| --- | --- | --- |
| 1 | 2 | 8 |

46.2.2.1. Se sim: Porquê?

46.2.2.2. Se não: Porque não?

46.2.3. E na sua saúde?

| Sim | Não | NS/NR |
| --- | --- | --- |
| 1 | 2 | 8 |

46.2.3.1. Se sim: Porquê?

46.2.3.2. Se não: Porque não?

**SECÇÃO I – PLANO DE CONTINGÊNCIA DE ONDAS DE CALOR**

47. Sabe da existência do Plano de Contigência de Ondas de Calor?

| Sim | Não | NS/NR |
| --- | --- | --- |
| 1 | 2 | 8 |

47.1. Se sim: 47.1.1. Quão importante acha que é?

| Muito importante | Importante | Nem muito nem pouco importante | Pouco importante | Nada importante |
| --- | --- | --- | --- | --- |
| 5 | 4 | 3 | 2 | 1 |

47.1.2. O que sabe acerca do Plano de Contigência de Ondas de Calor?

47.1.3. Gostaria de saber mais?

| Sim | Não | NS/NR |
| --- | --- | --- |
| 1 | 2 | 8 |

47.1.3.1. Se sim: O que gostaria de saber mais?

47.2. Se não: Gostaria de saber acerca do Plano de Contigência de Ondas de Calor?

| Sim | Não | NS/NR |
| --- | --- | --- |
| 1 | 2 | 8 |

47.2.1. Se sim: O que gostaria de saber?

48. Existe alguma coisa que gostaria de acrescentar ou dizer sobre o que fazer durante o tempo muito quente?

FIM DA ENTREVISTA

Obrigada pelo tempo dispensado para participar nesta entrevista. Tem alguma pergunta resultante desta entrevista?
Pergunte ao participante se ele/ela pode ser contactado novamente durante o inverno.

| Sim | Não | NS/NR |
| --- | --- | --- |
| 1 | 2 | 8 |

*[Informe o participante que o gravador foi desligado e já não está a gravar.]*

# Protocolo de Entrevista da Fase 2

**PROTOCOLO DE ENTREVISTA DE FRIO**

**Vou fazer-lhe algumas perguntas sobre si, a sua saúde e bem-estar, o seu bairro e a sua casa. Eu tenho que fazer as perguntas exactamente da mesma maneira a todos os participantes por isso pode parecer um pouco formal, mas diga apenas o que você pensa. Não existem respostas certas ou erradas a estas perguntas. Lembre-se que é livre de terminar esta entrevista a qualquer momento, diga-me apenas que deseja parar. Além disso, se existir uma questão em particular que não queira responder, por favor avise-me. Tem algumas questões antes de começar?** *[Informar o participante que o gravador foi ligado e está a gravar]*

**PARTE I – Atualização de informações desde a entrevista de verão**

**Gostaria de lhe perguntar se existem algumas alterações relacionadas consigo, a sua saúde e qualidade de vida, os seus contatos, o seu bairro e a sua casa desde que conversamos no verão. Por exemplo em matéria de:**

- Informação sócio-demográfica: estado civil; condições de vida
- Estado de saúde
- Qualidade de vida: padrão de vida, alojamento, vida familiar, saúde, vida social
- Relações sociais: filhos, família, amigos e vizinhos
- Características da habitação
- Características do bairro e vizinhança
- Rendimento e situação financeira - pagar despesas com: a habitação (aluguer, prestação da casa, eletricidade, água, gás), alimentação, cuidados de saúde ou medicamentos

* Como é que se preparou para este inverno? *(vacina da gripe?)*

* Considera que as suas atividades sociais e de lazer se alteram do verão para o inverno? ***Se sim:*** *Porquê? De que forma?*

**PARTE II** – **FRIO**

**SECÇÃO A – EXPERIÊNCIAS DE CALOR, PRÁTICAS QUOTIDIANAS E RESPOSTAS AO FRIO**

**Agora vou fazer-lhe algumas perguntas sobre como se sente o que faz quando o tempo está muito frio. No final vou também fazer-lhe algumas perguntas sobre a sua casa e sua saúde durante o tempo muito quente.**
1. Como acha que o tempo está hoje?

| Muito frio | Frio | Nem frio nem quente | Quente | Muito quente | NS/NR |
| --- | --- | --- | --- | --- | --- |
| 1 | 2 | 3 | 4 | 5 | 8 |

2. Quando foi a última vez que esteve muito frio nos últimos anos?

3. Onde estava nesse momento?

4. Houve alguma coisa que tenha começado a fazer para se proteger do tempo muito frio?

| Sim | Não | NS/NR |
| --- | --- | --- |
| 1 | 2 | 8 |

4.1. **Se sim:** O que começou a fazer? Em sua casa? Na rua? (Coisas relacionadas com a sua casa? Coisas relacionadas com o que veste? Coisas relacionadas com o que come?)

5. Houve alguma coisa que quisesse fazer mas não pudesse fazer?

| Sim | Não | NS/NR |
| --- | --- | --- |
| 1 | 2 | 8 |

5.1. **Se sim:** O quê?

Porque é que não pôde fazer?

6. Quando está muito frio, como se mantém quente em sua casa durante dia?

O que faz? Onde vai?

7. E durante a noite?

O que faz? Onde vai?

8. Quando está muito frio, como se mantém quente na rua?

O que faz? Onde vai?

9. Acha que é mais afetado(a) pelo tempo muito frio que as outras pessoas?

| Sim | Não | NS/NR |
| --- | --- | --- |
| 1 | 2 | 8 |

9.1. **Se sim:** Pode dizer-me porquê? De que maneira?

10. Consegue pensar em quaisquer grupos de pessoas que possam ser mais afetados pelo tempo muito frio?

| Sim | Não | NS/NR |
| --- | --- | --- |
| 1 | 2 | 8 |

10.1. **Se sim:** Em quem está a pensar? Pode dizer-me porquê?

11. Consegue pensar em algumas formas que o tempo muito frio possa afectar a saúde das pessoas?

| Sim | Não | NS/NR |
| --- | --- | --- |
| 1 | 2 | 8 |

11.1. **Se sim:** Pode falar um pouco mais sobre isso?

12. O tempo muito frio impede-o(a) de fazer as coisas que costuma fazer na sua vida diária?

| Sim | Não | NS/NR |
| --- | --- | --- |
| 1 | 2 | 8 |

12.1.**Se sim:** Porquê?

Quais as coisas que gostaria de fazer, mas não pode por causa do tempo muito frio?

**SECTION B –INFORMAÇÃO**

13. Recebeu alguma informação ou conselho sobre o que fazer durante o tempo muito frio?

| Sim | Não | NS/NR |
| --- | --- | --- |
| 1 | 2 | 8 |

13.1. **Se sim:** Foi você que pediu essa informação ou conselho?

| Sim | Não | NS/NR |
| --- | --- | --- |
| 1 | 2 | 8 |

13.1.1. **Se sim:** A1) A quem pediu informação ou conselho?
             A2) O que lhe disseram?
               A3) Quando / Onde?
                  A4) Foi útil? De que maneira?
            
              13.1.2. **Se não:** B1) Quem lhe deu a informação ou o conselho?
               B2) O que lhe disseram?
                                                                           B3) Quando / Onde?
                                                                        B4) Por que acha que lhe foi dada essa informação ou conselho?
                                                                           B5) Foi útil? De que maneira?

14. Forneceu alguma informação ou conselho a alguém sobre o que fazer durante o tempo muito frio**?**

| Sim | Não | NS/NR |
| --- | --- | --- |
| 1 | 2 | 8 |

14.1. **Se sim:** A quem? O que disse?

15. Quanta informação acha que tem?

| Muita | Alguma | Pouca | NS/NR |
| --- | --- | --- | --- |
| 3 | 2 | 1 | 8 |

16. Gostaria de ter (mais) informação ou conselhos sobre o que fazer durante o tempo muito frio?

| Sim | Não | NS/NR |
| --- | --- | --- |
| 1 | 2 | 8 |

16.1. **Se sim:** Que fonte(s) de informação preferiria?

| Contactos sociais (família, amigos, vizinhos) | Professional de saúde | Rádio | TV | Jornal | Internet | Outra | NS/NR |
| --- | --- | --- | --- | --- | --- | --- | --- |
| 1 | 2 | 3 | 4 | 5 | 6 | 7 | 8 |

16.2. Porquê?

17. Acha que o tempo está a ficar mais frio de ano para ano?

| Sim | Não | NS/NR |
| --- | --- | --- |
| 1 | 2 | 8 |

17.1. **Se sim:** Pode falar um pouco mais sobre isso?

**SECTION C –CONTACTOS SOCIAIS E SAÚDE DURANTE O TEMPO MUITO FRIO**

**Agora vou perguntar-lhe sobre a sua família e amigos.**

18. Com que frequência tem contacto com outras pessoas durante o tempo muito frio?

| Mais de uma vez por dia | Todos os dias ou quase todos os dias | Pelo menos uma vez por semana | Uma ou duas vezes por mês | Menos frequentemente | NS/NR |
| --- | --- | --- | --- | --- | --- |
| 5 | 4 | 3 | 2 | 1 | 8 |

19. Quando está preocupado ou necessita de alguma ajuda durante o tempo muito frio, a quem pede ajuda ou em quem pode confiar? *[anotar a relação com o participante]*

**Agora vou perguntar-lhe sobre a sua saúde**

20. A sua saúde física limita o que pode fazer durante o tempo muito frio?

| Sim | Não | NS/NR |
| --- | --- | --- |
| 1 | 2 | 8 |

21. O que faria se se sentisse mal/doente durante o tempo muito frio? *(Mais alguma coisa?)*

**SECTION D – CARACTERÍSTICAS DA RESIDÊNCIA**

**Apenas algumas perguntas sobre a sua casa durante o inverno**

22. Considera que a temperatura dentro de sua casa no Inverno é um problema?

| Sim | Não | NS/NR |
| --- | --- | --- |
| 1 | 2 | 8 |

23. Consegue manter-se quente em casa durante o inverno?

| Sim | Não | NS/NR |
| --- | --- | --- |
| 1 | 2 | 8 |

**Se sim:** Como?

**Se não:** Porque não?

24. Consegue manter a casa quente durante o inverno?

| Sim | Não | NS/NR |
| --- | --- | --- |
| 1 | 2 | 8 |

- 24.1. **Se sim:** Como mantém a casa quente? *(sistema de aquecimento fixo, dispositivos de aquecimento e fontes de calor? Quais as fontes de energia que eles usam? vidros duplos ou simples?*
- 24.2. **Se não**: 24.2.1. Porque não?

24.2.2.Acha que sentir/ter frio em casa tem efeitos na sua vida em geral?

| Sim | Não | NS/NR |
| --- | --- | --- |
| 1 | 2 | 8 |

24.2.2.1. **Se sim:** Porquê?

24.2.2.2. **Se não:** Porque não?

24.2.3. E na sua saúde?

| Sim | Não | NS/NR |
| --- | --- | --- |
| 1 | 2 | 8 |

24.2.3.1.**Se sim:** Porquê?

24.2.3.2. **Se não:** Porque não?

25. Nos últimos 2 anos, teve dificuldades em pagar as despesas de aquecimento?

| Sim, frequentemente | Sim, as vezes | Não, nunca | NS/NR |
| --- | --- | --- | --- |
| 1 | 2 | 3 | 8 |

**SECÇÃO E – PLANO DE CONTINGÊNCIA PARA AS VAGAS DE FRIO**

26. Sabe da existência do Plano de Contigência para as Vagas de Frio?

| Sim | Não | NS/NR |
| --- | --- | --- |
| 1 | 2 | 8 |

- 26.1. **Se sim:** Quão importante acha que é?

| Muito importante | Importante | Nem muito nem pouco importante | Pouco importante | Nada importante |
| --- | --- | --- | --- | --- |
| 1 | 2 | 3 | 4 | 5 |

26.1.2. O que sabe acerca do Plano de Contigência para as Vagas de Frio?

26.1.3. Gostaria de saber mais?

| Sim | Não | NS/NR |
| --- | --- | --- |
| 1 | 2 | 8 |

26.1.3.1. **Se sim:** O que gostaria de saber mais?

- 26.2. **Se não:** Gostaria de saber acerca do Plano de Contigência de Vagas de Frio?

| Sim | Não | NS/NR |
| --- | --- | --- |
| 1 | 2 | 8 |

26.2.1. **Se sim:** O que gostaria de saber?

**SECÇÃO F – PERCEÇÕES DE ADAPTAÇÃO E RESILIÊNCIA (tempo muito frio e muito quente)**

**Agora gostaria de lhe perguntar sobre a forma como lida com o tempo muito frio**

27. Como sente que lida com o tempo muito frio? *(Mais alguma coisa?)*

28. Existe alguma coisa que você **possa** fazer para melhorar a forma como lida com o tempo muito frio?

| Sim | Não | NS/NR |
| --- | --- | --- |
| 1 | 2 | 8 |

*Se sim:* O quê? Já fez isso? *(Mais alguma coisa?)*

*Se não:* Porque não? *Alguma coisa relacionada com a sua casa, alimenta*ção*, vestu*á*rio?*

29. Existe alguma coisa que você **gostasse** de fazer para melhorar a forma como lida com o tempo muito frio?

| Sim | Não | NS/NR |
| --- | --- | --- |
| 1 | 2 | 8 |

*Se sim:* O quê? Já fez isso? *(Mais alguma coisa?)*

*Se não:* Porque não? *Alguma coisa relacionada com a sua casa, alimenta*ção*, vestu*á*rio?*

30. Existe alguma que **possa** ser feito para si para melhorar a forma como lida com o tempo muito frio?

| Sim | Não | NS/NR |
| --- | --- | --- |
| 1 | 2 | 8 |

*Se sim:* O quê? Já fez isso? *(Mais alguma coisa?)*

*Se não:* Porque não? *Alguma coisa relacionada com a sua casa, alimenta*ção*, vestu*á*rio?*

31. Existe alguma coisa que você **gostasse** que fosse feito para si para melhorar a forma como lida com o tempo muito frio?

| Sim | Não | NS/NR |
| --- | --- | --- |
| 1 | 2 | 8 |

*Se sim:* O quê? Já fez isso? *(Mais alguma coisa?)*

*Se não:* Porque não? *Alguma coisa relacionada com a sua casa, alimenta*ção*, vestu*á*rio?*

32. Acha que **tem *(terá)*** os meios/recursos para ser capaz de lidar com o tempo muito frio **agora**? E no **futuro**?

| Sim | Não | NS/NR |
| --- | --- | --- |
| 1 | 2 | 8 |

| Sim | Não | NS/NR |
| --- | --- | --- |
| 1 | 2 | 8 |

*Se sim:* Porquê? Quais? *(Mais alguma coisa?)*

*Se não:* Porque não?

33. Existe alguma coisa que **possa** melhorar os meios/recursos para ser capaz de lidar com o tempo muito frio **agora**? E no **futuro**?

| Sim | Não | NS/NR |
| --- | --- | --- |
| 1 | 2 | 8 |

| Sim | Não | NS/NR |
| --- | --- | --- |
| 1 | 2 | 8 |

Feito pelo próprio ou por outros? *Se sim:* Porquê? O quê? *(Mais alguma coisa?)*

*Se não:* Porque não?

**Agora vou perguntar-lhe sobre a forma como lida com o tempo muito quente**

34. Como sente que lida com o tempo muito quente? *(Mais alguma coisa?)*

35.A) Existe alguma coisa que você **possa** fazer para melhorar a forma como lida com o tempo muito quente?

| Sim | Não | NS/NR |
| --- | --- | --- |
| 1 | 2 | 8 |

*Se sim:* O quê? Já fez isso? *(Mais alguma coisa?)*

*Se não:* Porque não? *Alguma coisa relacionada com a sua casa, alimenta*ção*, vestu*á*rio?*

36. Existe alguma coisa que você **gostasse** de fazer para melhorar a forma como lida com o tempo muito quente?

| Sim | Não | NS/NR |
| --- | --- | --- |
| 1 | 2 | 8 |

*Se sim:* O quê? Já fez isso? *(Mais alguma coisa?)*

*Se não:* Porque não? *Alguma coisa relacionada com a sua casa, alimenta*ção*, vestu*á*rio?*

37. Existe alguma que **possa** ser feito para si para melhorar a forma como lida com o tempo muito quente?

| Sim | Não | NS/NR |
| --- | --- | --- |
| 1 | 2 | 8 |

*Se sim:* O quê? Já fez isso? *(Mais alguma coisa?)*

*Se não:* Porque não? *Alguma coisa relacionada com a sua casa, alimenta*ção*, vestu*á*rio?*

38. Existe alguma coisa que você **gostasse** que fosse feito para si para melhorar a forma como lida com o tempo muito quente?

| Sim | Não | NS/NR |
| --- | --- | --- |
| 1 | 2 | 8 |

*Se sim:* O quê? Já fez isso? *(Mais alguma coisa?)*

*Se não:* Porque não? *Alguma coisa relacionada com a sua casa, alimenta*ção*, vestu*á*rio?*

39. Acha que tem ***(terá)*** os meios/recursos para ser capaz de lidar com o tempo muito quente **agora**? E no **futuro**?

| Sim | Não | NS/NR |
| --- | --- | --- |
| 1 | 2 | 8 |

| Sim | Não | NS/NR |
| --- | --- | --- |
| 1 | 2 | 8 |

*Se sim:* Porquê? Quais?

*(Mais alguma coisa?)*

*Se não:* Porque não?

40. Existe alguma coisa que **possa** melhorar os meios/recursos que tem para ser capaz de lidar com o tempo muito quente **agora**?

| Sim | Não | NS/NR |
| --- | --- | --- |
| 1 | 2 | 8 |

| Sim | Não | NS/NR |
| --- | --- | --- |
| 1 | 2 | 8 |

E no **futuro**?

*Se sim:* Porquê? O quê? *(Mais alguma coisa?)*

*Se não:* Porque não?

Feito pelo próprio ou por ‘outros’?

41. Qual e a sua opinião sobre como pessoalmente lida com o tempo muito frio ou muito quente? *(Mais alguma coisa?)*

42. Gostaria de acrescentar mais alguma coisa?

**FIM DA ENTREVISTA**

Obrigada por ter disponibilizado o seu e tempo e ter participado nesta entrevista. Tem alguma duvida decorrente da entrevista?
*Informe o participante que o gravador foi desligado e já não está a gravaR*
