# Supplementary material for: The contribution of assets to adaptation to extreme temperatures among older adults
Source: PLoS One. 2018 Nov 29;13(11):e0208121. doi: 10.1371/journal.pone.0208121 (PMC6264854; doi:10.1371/journal.pone.0208121)
Supplement: S2 File — (DOCX) [file pone.0208121.s003.docx]

**Phase 1 and 2 Interview Protocols: English Translation of Original Portuguese Text**

**GENERAL STRUCTURED AND HEAT-RELATED SEMI-STRUCTURED INTERVIEW PROTOCOL**

**PHASE 1 – GENERAL AND EXTREME HEAT**

**SECTION A - SOCIO-DEMOGRAPHIC INFORMATION**

1. Record sex as observed: Female (1), Male (2)

2. What is your age? ___ (years)

3. What is your current marital status?

| Married | Divorced | Widowed | Single |
| --- | --- | --- | --- |
| 4 | 3 | 2 | 1 |

4. Do you live with...?

| Spouse | Family members (e.g. children, brothers, sisters, etc) | Other non relatives (e.g. friends, colleagues, etc.) | Alone |
| --- | --- | --- | --- |
| 4 | 3 | 2 | 1 |

5. What is the highest level of education you have successfully completed?

| elementary education | 1 |
| --- | --- |
| middle school | 2 |
| high school | 3 |
| college | 4 |
| graduate school | 5 |

*(The response categories will be named according to the Portuguese educational system)*

**SECTION B – HEALTH**

Now I’m going to ask you about your health, wellbeing and your life

6. In general, would you say your health is:

| Excellent | Very good | Good | Fair | Poor |
| --- | --- | --- | --- | --- |
| 5 | 4 | 3 | 2 | 1 |

7. Compared to one year ago, how would you rate your health in general now?

| Much better now | Somewhat better now | About the same | Somewhat worse | Much worse |
| --- | --- | --- | --- | --- |
| 5 | 4 | 3 | 2 | 1 |

8. Are you hampered in your daily activities in any way by any longstanding illness, or disability, infirmity, mobility problem or mental health problem?

| Yes, a lot | Yes, to some extent | No | [DK/N A] |
| --- | --- | --- | --- |
| 1 | 2 | 3 | 8 |

9.1. If yes: Which? ________________________________

**SECTION C - HOUSING and APPLIANCES INFORMATION**

Just a few questions about your home and neighbourhood

9. What best describes the house you live in?

| Detached single-family house | Semi-detached house or duplex | Terraced housing unit | Apartment building with up to 3 storeys | Apartment building with 4 to 8 storeys | Apartment building with 9 or more storeys |
| --- | --- | --- | --- | --- | --- |
| 1 | 2 | 3 | 4 | 5 | 6 |

10. What floor do you live on? _______

11. Is there a lift in the building?

| No | Yes, and it works | Yes, but it doesn’t work | [DK/N A] |
| --- | --- | --- | --- |
| 1 | 2 | 3 | 8 |

12. What is the age of the building or house you live in?

| Under 10 years | 10-19 | 20-29 | 30-39 | 40-49 | 50-59 | 60-74 | 75-99 | 100 and more | [DK/N A] |
| --- | --- | --- | --- | --- | --- | --- | --- | --- | --- |
| 1 | 2 | 3 | 4 | 5 | 6 | 7 | 8 | 9 |  |

13. On a scale from 1 to 5, how satisfied are you with your house?

| Highly dissatisfied |  |  |  | Highly satisfied |
| --- | --- | --- | --- | --- |
| 1 | 2 | 3 | 4 | 5 |

14. Is the house you live in ...?

| Owned | Rented | Social housing |
| --- | --- | --- |
| 1 | 2 | 3 |

15. Are you happy with your current living conditions or would you move if you had the chance?

| Am happy | Would move |
| --- | --- |
| 1 | 2 |

15.1. If would move: Why would you move? ______________

16. Which of the following equipments/goods do you have? 16.7. If yes: Do you use them?

|  | Yes | No |  | Yes | No |
| --- | --- | --- | --- | --- | --- |
| 16.1. Fixed telephone | 1 | 2 |  | 1 | 2 |
| 16.2. Mobile telephone |  |  |  |  |  |
| 16.3. Television |  |  |  |  |  |
| 16.4. Radio |  |  |  |  |  |
| 16.5. Computer |  |  |  |  |  |
| 16.6. Car |  |  |  |  |  |

**SECTION D - FINANCE/INCOME**

17. How would you describe the current financial situation of your household?

| Very comfortable | Comfortable | We have to be careful, but we manage | We have difficulties making ends meet | Things are very difficult | [DK/N A] |
| --- | --- | --- | --- | --- | --- |
| 5 | 4 | 3 | 2 | 1 | 8 |

18. Please can you tell me how much your household’s average income per month is (after taxes)? If you don’t know the exact figure, please give an estimate.

| ≤ 350 euros | 351-500 | 501-800 | 801-1500 | 1501-2500 | > 2500 euros | [DK/N A] |
| --- | --- | --- | --- | --- | --- | --- |
| 1 | 2 | 3 | 4 | 5 | 6 | 8 |

19. Have you or someone else in your household received any of the following types of income over the past 12 months?

|  | Yes | No | [DK/N A] |
| --- | --- | --- | --- |
| 19.1. Wages or salaries | 1 | 2 | 8 |
| 19.2. Income from self-employment or farming |  |  |  |
| 19.3. Pension |  |  |  |
| 19.4. Unemployment benefit, disability benefit or any other social benefits |  |  |  |
| 19.5. Other income (e.g. from savings, property or stocks, etc.) |  |  |  |

20. In the past 2 years, have there been difficulties paying the housing expenses? (i.e. rent or mortgage payments for accommodation, utility bills, such as electricity, water, gas)

| No, never | Yes, sometimes | Yes, often | [DK/N A] |
| --- | --- | --- | --- |
| 3 | 2 | 1 | 8 |

If yes: 20.1. Which ones? ____________________ 20.2. Why?___________________________

21. Has your household at any time during the past 12 months had difficulties in paying for food?

| Yes | No | [DK/N A] |
| --- | --- | --- |
| 1 | 2 | 8 |

35.1. If yes: Can you tell me a bit more about it?

22. Has your household at any time during the past 12 months had difficulties in paying for healthcare or medication?

| Yes | No | [DK/N A] |
| --- | --- | --- |
| 1 | 2 | 8 |

22.1. If yes: Can you tell me a bit more about it?

23. If for some reason you were in serious financial difficulties and had to borrow money to make ends meet, how difficult or easy would that be?

| Very difficult | Quite difficult | Neither easy nor difficult | Quite easy | Very easy | [DK/N A] |
| --- | --- | --- | --- | --- | --- |
| 1 | 2 | 3 | 4 | 5 | 8 |

23.1. Why? _______________________________________________________________________

23.2. Whom would you ask for money? *[note relationship with participant]____________________*

**EXTREME HEAT INFORMATION**

**SECTION E – EXPERIENCES OF HEAT, EVERYDAY BEHAVIOURS AND RESPONSES TO HEAT**

Now I am going to ask you some questions about how you feel and what you do when the weather is very hot. At the end I’ll also ask you a few questions about your home and your health during very hot weather.

24. When was the last time it was very hot in recent years?

25. Where were you at the time?

26. Was there anything you started doing to protect yourself from very hot weather?

| Yes | No | [DK/N A] |
| --- | --- | --- |
| 1 | 2 | 8 |

26.1. If yes: What did you start doing? Inside your home? When you’re outside? (Things to do with your house? Things to do with what you wear? Things to do with what you eat?)

27. Was there anything you wanted to do but were unable to do?

| Yes | No | [DK/N A] |
| --- | --- | --- |
| 1 | 2 | 8 |

27.1. If yes: What?

Why were you unable to do it?

28. When it’s very hot how do you keep cool inside your house during the day?

What do you do?

Where do you go?

29. And during the night?

What do you do?

Where do you go?

30. When it’s very hot how do you keep cool outside?

What do you do?

Where do you go?

31. Do you think you’re more affected by very hot weather than other people?

| Yes | No | [DK/N A] |
| --- | --- | --- |
| 1 | 2 | 8 |

31.1. If yes: Can you tell me why? In what way?

32. Can you think of any other groups of people who may be more affected by very hot weather?

| Yes | No | [DK/N A] |
| --- | --- | --- |
| 1 | 2 | 8 |

32.1. If yes: Who are you thinking about? Can you tell me why?

33. Can you think of any ways that very hot weather can affect people’s health?

| Yes | No | [DK/N A] |
| --- | --- | --- |
| 1 | 2 | 8 |

33.1. If yes: Can you say a bit more about that?

34. Does very hot weather stop you from doing the things you usually do in your everyday life?

| Yes | No | [DK/N A] |
| --- | --- | --- |
| 1 | 2 | 8 |

34.1. If yes: Why?

What things would you like to do but can’t because of very hot weather?

**SECTION F –INFORMATION**

35. Have you received any information, or advice on what to do during very hot weather?

| Yes | No | [DK/N A] |
| --- | --- | --- |
| 1 | 2 | 8 |

35.1. If yes: Did you ask for this information, advice or support?

| Yes | No | [DK/N A] |
| --- | --- | --- |
| 1 | 2 | 8 |

35.1.1. If yes: A1) Whom did you ask for information or advice?

A2) What did they say?

A3) When / Where?

A4) Was it helpful? In what way?

35.1.2. If no: B1)Who gave you the information or advice?

B2) What did they say?

B3) When / Where?

B4) Why do you think you were given that information or advice?

B5) Was it helpful? In what way?

36. Have you provided any information or advice to someone about what to do during very hot weather?

| Yes | No | [DK/N A] |
| --- | --- | --- |
| 1 | 2 | 8 |

36.1. If yes: To whom?

What did you say?

37. How much information do you think you have about what to do during very hot weather?

| Much | Some | Little | [DK/N A] |
| --- | --- | --- | --- |
| 3 | 2 | 1 | 8 |

38. Would you like to have more information or advice about what to do during very hot weather?

| Yes | No | [DK/N A] |
| --- | --- | --- |
| 1 | 2 | 8 |

38.1. If yes: What source(s) of information would you prefer?

| Social contacts (family, friends, neighbours) | Health professional | Radio | TV | Newspaper | Internet | Other | [DK/N A] |
| --- | --- | --- | --- | --- | --- | --- | --- |
| 1 | 2 | 3 | 4 | 5 | 6 | 7 | 8 |

38.2. Why?

39. Do you think the weather is getting hotter year on year?

| Yes | No | [DK/N A] |
| --- | --- | --- |
| 1 | 2 | 8 |

39.1. If yes: Can you say a bit more about that?

**SECTION G – SOCIAL CONTACTS AND HEALTH DURING VERY HOT WEATHER**

Now I’m going to ask you about your family and friends during very hot weather.

40. How often do you have contact with other people during very hot weather?

| More than once a day | Every day or almost everyday | At least once a week | Once or twice a month | Less often | [DK/N A] |
| --- | --- | --- | --- | --- | --- |
| 5 | 4 | 3 | 2 | 1 | 8 |

41. When you’re concerned or need help with anything during very cold weather who do you ask and rely on? *[note relationship with participant]*

Now I’m going to ask you about your health during very hot weather.

42. Does your physical health limit what you can do during very hot weather?

| Yes | No | [DK/N A] |
| --- | --- | --- |
| 1 | 2 | 8 |

42.1. If yes: Why is that?

In what way?

43. What would you do if you felt unwell during very hot weather? *(Anything else?)*

**SECTION H – RESIDENTIAL CHARACTERISTICS**

Just a few questions about your home during summer

44. Do you perceive the temperature in your home during the summer season as a problem?

| Yes | No | [DK/N A] |
| --- | --- | --- |
| 1 | 2 | 8 |

45. Are you able to keep cool in your house during summer?

| Yes | No | [DK/N A] |
| --- | --- | --- |
| 1 | 2 | 8 |

45.1. If yes: How?

45.2. If not: Why not?

46. Are you able to keep your house cool during summer?

| Yes | No | [DK/N A] |
| --- | --- | --- |
| 1 | 2 | 8 |

46.1. If yes: How is your house kept cool?

46.2. If not: 46.2.1. Why not?

46.2.2. Do you think that being hot at home has effects on your life in general?

| Yes | No | [DK/N A] |
| --- | --- | --- |
| 1 | 2 | 8 |

46.2.2.1.If yes: Why?

46.2.2.2. If not: Why not?

46.2.3. And on your health?

| Yes | No | [DK/N A] |
| --- | --- | --- |
| 1 | 2 | 8 |

46.2.3.1. If yes: Why?

46.2.3.2. If not: Why not?

**SECTION I – HEATWAVE PLAN**

47. Do you know about the Heatwave Plan?

| Yes | No | [DK/N A] |
| --- | --- | --- |
| 1 | 2 | 8 |

47.1. If yes: 47.1.1.How important do you think it is?

| Very important | Important | Moderately important | Of little importance | Unimportant |
| --- | --- | --- | --- | --- |
| 5 | 4 | 3 | 2 | 1 |

47.1.2. What do you know about the Heatwave Plan?

47.1.3. Would you like to know more?

| Yes | No | [DK/N A] |
| --- | --- | --- |
| 1 | 2 | 8 |

47.1.3.1. If yes: What would you like to know more?

47.2. If no: Would you like to know about the Heatwave Plan?

| Yes | No | [DK/N A] |
| --- | --- | --- |
| 1 | 2 | 8 |

65.2.1. If yes: What would you like to know?

48. Is there anything else you’d like to add or say about coping during very hot weather?

**END OF INTERVIEW**

Thank you for taking the time to participate in this interview. Do you have further questions arising from this interview? Ask the participant if he/her can be contacted again during winter season.

| Yes | No | [DK/N A] |
| --- | --- | --- |
| 1 | 2 | 8 |

*[Notify the participant that the recorder has been switched off and is no longer recording*.*]*

# Phase 2 Interview Protocol

**EXTREME COLD SEMI-STRUCTURED INTERVIEW PROTOCOL**

**In the summer we spoke about very hot weather and about you, your health and wellbeing, your neighbourhood and your home. Today I would like to ask questions about very cold weather.**

**I will read you the questions out loud and it may sound a bit formal but I need to ask them in the same way to other participants. Please let me know what you think, as there is no right or wrong answers. Also remember that you can withdraw from this interview at any time, by letting me know you wish to stop. In addition, if there is a particular question you do not wish to answer, please let me know. Do you have any questions before we start?**

*[At this point inform the participant that the recorder has been switched on and is recording.]*

**PART I – Updated information since summer interview**

**I would like to ask you if there were any changes related to you, your health and quality of life, your social contacts and activities, your neighbourhood and your home since we talked in the summer. For example concerning:**

- Socio-demographic information: marital status; living arrangements
- Health status
- Quality of Life: standard of living; accommodation; family life; health; social life
- Social contacts: children, family, friends and neighbours
- Housing characteristics
- Neighbourhood characteristics
- Income and financial situation: paying housing expenses (rent or mortgage, electricity, water, gas), food, healthcare or medication

* How have you prepared for winter? *(Flu jab?)*

* Do you think your social and leisure activities change from summer to winter? ***If yes:*** *Why is that? In what way?*

**PART II – COLD**

**SECTION A – EXPERIENCES OF COLD, EVERYDAY BEHAVIOURS AND RESPONSES TO COLD**

**Now I am going to ask you some questions about how you feel and what you do when the weather is very cold. At the end I’ll also ask you a few questions about your home and your health during very cold weather.**

1. How do you think the weather is today?

| Very cold | Cold | Neither cold nor warm | Warm | Very warm | [DK/N A] |
| --- | --- | --- | --- | --- | --- |
| 1 | 2 | 3 | 4 | 5 | 8 |

2. When was the last time it was very cold in recent years?

3. Where were you at the time?

4. Was there anything you started doing to protect yourself from very cold weather?

| Yes | No | [DK/N A] |
| --- | --- | --- |
| 1 | 2 | 8 |

**4.1. If yes:** What did you start doing? Inside your home? When you’re outside? (Things to do with your house? Things to do with what you wear? Things to do with what you eat?)

5. Was there anything you wanted to do but were unable to do to cope with very cold weather?

| Yes | No | [DK/N A] |
| --- | --- | --- |
| 1 | 2 | 8 |

**5.1. If yes:** What?

Why were you unable to do it?

6. When it’s very cold how do you keep warm inside your house during the day?

What do you do? Where do you go?

7. And during the night?

What do you do? Where do you go?

8. When it’s very cold how do you keep warm outside?

What do you do? Where do you go?

9. Do you think you’re more affected by very cold weather than other people?

| Yes | No | [DK/N A] |
| --- | --- | --- |
| 1 | 2 | 8 |

9.1. **If yes:** Can you tell me why? In what way?

10. Can you think of any groups of people who may be more affected by very cold weather?

| Yes | No | [DK/N A] |
| --- | --- | --- |
| 1 | 2 | 8 |

10.1 **If yes:** Who are you thinking about?

Can you tell me why?

11. Can you think of any ways that very cold weather can affect people’s health?

| Yes | No | [DK/N A] |
| --- | --- | --- |
| 1 | 2 | 8 |

11.1. **If yes:** Can you say a bit more about that?

12. Does very cold weather stop you from doing the things you usually do in your everyday life?

| Yes | No | [DK/N A] |
| --- | --- | --- |
| 1 | 2 | 8 |

12.1. **If yes:** Why?

What things would you like to do but can’t because of very cold weather? *(Please specify)*

**SECTION B –INFORMATION**

13. Have you received any information, or advice on what to do during very cold weather?

| Yes | No | [DK/N A] |
| --- | --- | --- |
| 1 | 2 | 8 |

13.1. **If yes:** Did you ask for this information, advice or support?

| Yes | No | [DK/N A] |
| --- | --- | --- |
| 1 | 2 | 8 |

13.1.1. **If yes:** A1) Whom did you ask for information or advice?

A2) What did they say?

A3) When / Where?

A4) Was it helpful? In what way?

13.1.2. **If no:** B1) Who gave you the information or advice?

B2) What did they say?

B3) When / Where?

B4) Why do you think you were given that information or advice?

B5) Was it helpful? In what way?

14. Have you provided any information or advice to someone about what to do during very cold weather?

| Yes | No | [DK/N A] |
| --- | --- | --- |
| 1 | 2 | 8 |

14.1**. If yes:** To whom?

15. How much information do you think you have about what to do during very cold weather?

| Much | Some | Little | [DK/N A] |
| --- | --- | --- | --- |
| 3 | 2 | 1 | 8 |

16. Would you like to have (more) information or advice about what to do during very cold weather?

| Yes | No | [DK/N A] |
| --- | --- | --- |
| 1 | 2 | 8 |

16.1. **If yes:** What source(s) of information would you prefer?

| Social contacts (family, friends, neighbours) | Health professional | Radio | TV | Newspaper | Internet | Other | [DK/N A] |
| --- | --- | --- | --- | --- | --- | --- | --- |
| 1 | 2 | 3 | 4 | 5 | 6 | 7 | 8 |

16.2. Why?

17. Do you think the weather is getting colder year on year?

| Yes | No | [DK/N A] |
| --- | --- | --- |
| 1 | 2 | 8 |

17.1. **If yes:** Can you say a bit more about that?

**SECTION C – SOCIAL CONTACTS AND HEALTH DURING VERY COLD WEATHER**

**Now I’m going to ask you about your family and friends.**

18. How often do you have contact with other people during very cold weather?

| More than once a day | Every day or almost everyday | At least once a week | Once or twice a month | Less often | [DK/N A] |
| --- | --- | --- | --- | --- | --- |
| 5 | 4 | 3 | 2 | 1 | 8 |

19. When you’re concerned or need help with anything during very cold weather who do you ask and rely on? *[note relationship with participant]*

**Now I’m going to ask you about your health**

20. Does your physical health limit what you can do during very cold weather?

| Yes | No | [DK/N A] |
| --- | --- | --- |
| 1 | 2 | 8 |

20.1. **If yes:** Why is that?

In what way?

21. What would you do if you felt unwell during very cold weather? *(Anything else?)*

**SECTION D – RESIDENTIAL CHARACTERISTICS**

**Just a few questions about your home during winter**

22. Do you perceive the temperature in your home during the winter season as a problem?

| Yes | No | [DK/N A] |
| --- | --- | --- |
| 1 | 2 | 8 |

23. Are you able to keep warm in your house during winter?

| Yes | No | [DK/N A] |
| --- | --- | --- |
| 1 | 2 | 8 |

**If yes:** How?

**If not:** Why not?

24. Are you able to keep your house warm during winter?

| Yes | No | [DK/N A] |
| --- | --- | --- |
| 1 | 2 | 8 |

- 24.1. **If yes:** How is your house kept warm? *(Do you use fixed or installed heating system, heating devices and heat sources? What energy sources do they use? Double or single glazing?)*
- 24.2. **If not:** 24.2.1. Why not? What are the reasons? *(Do you use fixed or installed heating system, heating devices and heat sources? What energy sources do they use? Double or single glazing? Cannot afford heating?)*

24.2.2. Do you think that being cold at home has effects on your life in general?

| Yes | No | [DK/N A] |
| --- | --- | --- |
| 1 | 2 | 8 |

24.2.2.1. **If yes:** Why?

24.2.2.2. **If not:** Why not?

24.2.3. And on your health?

| Yes | No | [DK/N A] |
| --- | --- | --- |
| 1 | 2 | 8 |

25.2.3.1. **If yes:** Why?

25.2.3.2. **If not:** Why not?

25. In the past 2 years, have there been difficulties paying the heating expenses?

| Yes, often | Yes, sometimes | No, never | [DK/N A] |
| --- | --- | --- | --- |
| 1 | 2 | 3 | 8 |

**SECTION E – COLD WEATHER PLAN**

26. Do you know about the Cold Weather Plan?

| Yes | No | [DK/N A] |
| --- | --- | --- |
| 1 | 2 | 8 |

- 26.1. **If yes:** 26.1.1. How important do you think it is?

| Very important | Important | Moderately important | Of little importance | Unimportant |
| --- | --- | --- | --- | --- |
| 5 | 4 | 3 | 2 | 1 |

26.1.2. What do you know about the Cold Weather Plan?

26.1.3. Would you like to know more?

| Yes | No | [DK/N A] |
| --- | --- | --- |
| 1 | 2 | 8 |

26.1.3.1. **If yes:** What would you like to know more?

- 26.2. **If no:** Would you like to know about the Cold Weather Plan?

| Yes | No | [DK/N A] |
| --- | --- | --- |
| 1 | 2 | 8 |

26.2.1. **If yes:** What would you like to know?

**SECTION F – PERCEPTIONS OF OWN ADAPTATION AND RESILIENCE (very cold and very hot weather)**

**The next questions focus on your views about very cold and very hot weather.**

27. How do you feel you cope/deal with very cold weather? *(Anything else?)*

28. Is there anything that you **could** do to improve the way you cope/deal with very cold weather?

| Yes | No | [DK/N A] |
| --- | --- | --- |
| 1 | 2 | 8 |

*If yes: What? Have you done it? (Anything else?)*

*If no: Why not? Anything to do with your house, clothing, nutrition?*

29. Is there anything that you **would like** to do to improve the way you cope/deal with very cold weather?

| Yes | No | [DK/N A] |
| --- | --- | --- |
| 1 | 2 | 8 |

*If yes: What? Have you done it? (Anything else?)*

*If no: Why not? Anything to do with your house, clothing, nutrition?*

30. Is there anything that **could** be done for you to improve the way you cope/deal with very cold weather?

| Yes | No | [DK/N A] |
| --- | --- | --- |
| 1 | 2 | 8 |

*If yes: What? (Anything else?)*

*If no: Why not? Anything to do with your house, clothing, nutrition?*

31. Is there anything that you **would like** to be done for you to improve the way you cope/deal with very cold weather?

| Yes | No | [DK/N A] |
| --- | --- | --- |
| 1 | 2 | 8 |

*If yes: What? (Anything else?)*

*If no: Why not? Anything to do with your house, clothing, nutrition?*

32. Do you think you *(will)* have the means/resources to be able to cope/deal with very cold weather **now?** And in the **future**?

| Yes | No | [DK/N A] |
| --- | --- | --- |
| 1 | 2 | 8 |

| Yes | No | [DK/N A] |
| --- | --- | --- |
| 1 | 2 | 8 |

*If yes: Why? Which?*

*If no: Why not?*

| Yes | No | [DK/N A] |
| --- | --- | --- |
| 1 | 2 | 8 |

33. Is there anything that **could** improve the means/resources you have to be able to cope/deal with very cold weather **now**?

| Yes | No | [DK/N A] |
| --- | --- | --- |
| 1 | 2 | 8 |

And in the **future**?

*If yes: Why? What?*

*If no: Why not? Anything to do with your house, clothing, nutrition?*

Done by oneself or by ‘others’?

34. How do you feel you cope/deal with very hot weather? *(Anything else?)*

35. Is there anything that you **could** do to improve the way you cope/deal with very hot weather?

| Yes | No | [DK/N A] |
| --- | --- | --- |
| 1 | 2 | 8 |

*If yes: What? Have you done it? (Anything else?)*

*If no: Why not? Anything to do with your house, clothing, nutrition?*

36. Is there anything that you **would like** to do to improve the way you cope/deal with very hot weather?

| Yes | No | [DK/N A] |
| --- | --- | --- |
| 1 | 2 | 8 |

*If yes: What? Have you done it? (Anything else?)*

*If no: Why not? Anything to do with your house, clothing, nutrition?*

37. Is there anything that **could** be done for you to improve the way you cope/deal with very hot weather?

| Yes | No | [DK/N A] |
| --- | --- | --- |
| 1 | 2 | 8 |

*If yes: What? (Anything else?)*

*If no: Why not? Anything to do with your house, clothing, nutrition?*

38. Is there anything that you **would like** to be done for you to improve the way you cope/deal with very hot weather?

| Yes | No | [DK/N A] |
| --- | --- | --- |
| 1 | 2 | 8 |

*If yes: What? (Anything else?)*

*If no: Why not? Anything to do with your house, clothing, nutrition?*

39. Do you think you *(will)* have the means/resources to be able to cope/deal with very hot weather **now?** And in the **future**?

| Yes | No | [DK/N A] |
| --- | --- | --- |
| 1 | 2 | 8 |

| Yes | No | [DK/N A] |
| --- | --- | --- |
| 1 | 2 | 8 |

*If yes: Why? Which? If no: Why not?*

40. Is there anything that **could** improve the means/resources you have to be able to cope/deal with very hot weather **now**?

| Yes | No | [DK/N A] |
| --- | --- | --- |
| 1 | 2 | 8 |

| Yes | No | [DK/N A] |
| --- | --- | --- |
| 1 | 2 | 8 |

And in the **future**?

*If yes: Why? What?*

*If no: Why not? Anything to do with your house, clothing, nutrition?*

Done by oneself or by ‘others’?

41. What are your views about how you personally cope/deal with very cold weather or very hot weather? *(Anything else?)*

42. Would you like to add anything else?

**END OF INTERVIEW**

Thank you for taking the time to participate in this interview. Do you have further questions arising from this interview?

*[Notify the participant that the recorder has been switched off and is no longer recording*.*]*
